# Bursts of vagus nerve stimulation paired with auditory rehabilitation fail to improve speech sound perception in rats with hearing loss

**DOI:** 10.1016/j.isci.2024.109527

**Published:** 2024-03-19

**Authors:** Alan M. Carroll, Jonathan R. Riley, Michael S. Borland, Tanya T. Danaphongse, Seth A. Hays, Michael P. Kilgard, Crystal T. Engineer

**Affiliations:** 1 The University of Texas at Dallas, Texas Biomedical Device Center, 800 West Campbell Road, Richardson, TX 75080-3021, USA; 2 Department of Neuroscience, School of Behavioral and Brain Sciences, The University of Texas at Dallas, 800 West Campbell Road, Richardson, TX 75080-3021, USA; 3 Department of Bioengineering, Erik Jonsson School of Engineering and Computer Science, The University of Texas at Dallas, 800 West Campbell Road, Richardson, TX 75080-3021, USA

**Keywords:** Neuroscience, Sensory neuroscience

## Abstract

Hearing loss can lead to long-lasting effects on the central nervous system, and current therapies, such as auditory training and rehabilitation, show mixed success in improving perception and speech comprehension. Vagus nerve stimulation (VNS) is an adjunctive therapy that can be paired with rehabilitation to facilitate behavioral recovery after neural injury. However, VNS for auditory recovery has not been tested after severe hearing loss or significant damage to peripheral receptors. This study investigated the utility of pairing VNS with passive or active auditory rehabilitation in a rat model of noise-induced hearing loss. Although auditory rehabilitation helped rats improve their frequency discrimination, learn novel speech discrimination tasks, and achieve speech-in-noise performance similar to normal hearing controls, VNS did not enhance recovery of speech sound perception. These results highlight the limitations of VNS as an adjunctive therapy for hearing loss rehabilitation and suggest that optimal benefits from neuromodulation may require restored peripheral signaling.

## Highlights

• Noise-induced hearing loss decreased speech discrimination performance in rats
• Rats received auditory rehabilitation paired with vagus nerve stimulation
• Training with vagus nerve stimulation failed to improve speech discrimination

## Graphical abstract

**Figure:**
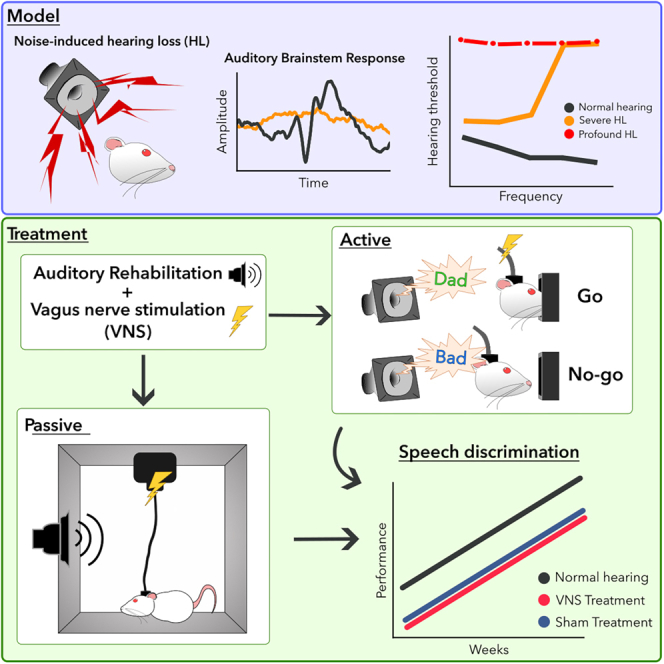

## Introduction

Hearing loss is one of the most prevalent disorders in the world, with recent estimates indicating nearly 500 million suffer globally.^1^ The burden of hearing loss extends well beyond decreased audibility of sounds. In addition to deficits in speech comprehension, poor auditory processing in the years following the onset of hearing loss is associated with social isolation, depression, declining cognitive function, and increased risk for dementia.^2^^,^^3^^,^^4^ Hearing aids are often the first step taken to augment remaining hearing ability. They are issued and worn with the anticipation that amplification or onboard signal processing will help to restore functional auditory capabilities in the user. While hearing aids can meaningfully increase the detection of low-volume sounds, they do not restore normal perception or sensitivity in noise, and may even make discrimination of some sounds more difficult,^5^^,^^6^^,^^7^^,^^8^ leading to many individuals choosing not to wear them at all.^6^^,^^9^ The limited treatment benefit highlights a well-documented but underappreciated fact: hearing loss is not simply a loss of sensitivity at the cochlea, but a dysfunction of the entire auditory processing chain, from periphery to cortex.^10^^,^^11^^,^^12^^,^^13^ While some changes in central auditory pathways may help compensate for reduced afferent input, recent evidence indicates that these changes can also be maladaptive, resulting in decreased spectral, spatial, and temporal resolutions, especially in noise.^3^^,^^5^^,^^11^^,^^12^^,^^14^^,^^15^^,^^16^^,^^17^^,^^18^ The physiological damage and subsequent neural adaptations following hearing loss are typically left untreated and are considered permanently altered.

No clinically available treatment can yet repair damage at the cochlea and auditory nerve. However, because deficits from hearing loss are at least partly due to maladaptive alterations of auditory circuits, it may be possible to improve outcomes in patients by reversing maladaptive plasticity through rehabilitation.^19^^,^^20^^,^^21^^,^^22^^,^^23^^,^^24^ Auditory training (AT) is a hearing loss rehabilitation strategy that attempts to improve auditory skills through active listening and perceptual learning.^25^ Enhancing auditory function and processing through training may help delay or even partially reverse the negative consequences of hearing loss. Although utilizing AT for the restoration of speech processing and auditory capabilities has been an ongoing topic of interest for decades,^13^^,^^26^^,^^27^ little progress has been made in implementing successful AT rehabilitation programs.^25^^,^^26^^,^^28^^,^^29^^,^^30^ As the population of those suffering from hearing loss continues to grow, it is vital that we seek improvements in AT outcomes and push development of novel auditory therapies.

Vagus nerve stimulation (VNS) is an FDA-approved adjunctive therapy technique for enhancing rehabilitative outcomes. Pairing VNS with rehabilitative training has consistently driven substantially better functional outcomes compared to rehabilitation alone in multiple pre-clinical models of disparate motor and sensory disorders. Brief bursts of VNS applied during motor or sensory rehabilitation can produce faster, greater, and better generalized long-lasting recovery compared to training without VNS in rat models of ischemic and hemorrhagic stroke, traumatic brain injury, peripheral nerve injury, spinal cord injury, post-traumatic stress disorder, tinnitus, and even Rett syndrome.^31^^,^^32^^,^^33^^,^^34^^,^^35^^,^^36^^,^^37^^,^^38^^,^^39^^,^^40^^,^^41^^,^^42^^,^^43^^,^^44^^,^^45^^,^^46^ In clinical populations, VNS paired with rehabilitation has likewise driven improvements in tinnitus and in sensory and motor deficits after stroke.^47^^,^^48^^,^^49^^,^^50^^,^^51^^,^^52^^,^^53^^,^^54^ These benefits derive from the ability of VNS to facilitate changes in neural circuits in the central nervous system through activation of neuromodulatory networks.^33^^,^^55^^,^^56^^,^^57^^,^^58^^,^^59^ It is possible, as observed in these other models of neural dysfunction, that pairing VNS with AT will reshape the auditory network engaged by training and produce improvements in functional outcomes after hearing loss.

Here, we tested in two separate experiments whether VNS paired with AT could increase auditory performance measures after intense noise exposure (NE)-induced hearing loss in rats compared to equivalent AT without VNS. In Experiment 1, rats underwent NE inducing moderate to severe hearing loss and were tested on tonal detection and discrimination thresholds before and after passive AT, followed by weeks of active AT while learning a novel speech discrimination task. In Experiment 2, rats were exposed to noise that induced severe to profound hearing loss, followed by testing on speech detection, discrimination, and speech discrimination in noise after weeks of passive AT. This is the first study to attempt to augment behavioral performance with VNS following moderate to profound hearing loss, and the first to utilize an adjunctive VNS therapy for rehabilitation after significant long-term damage to peripheral receptors.

## Results

### Experiment 1

#### Noise exposure increases ABR thresholds

**Figure 1:**
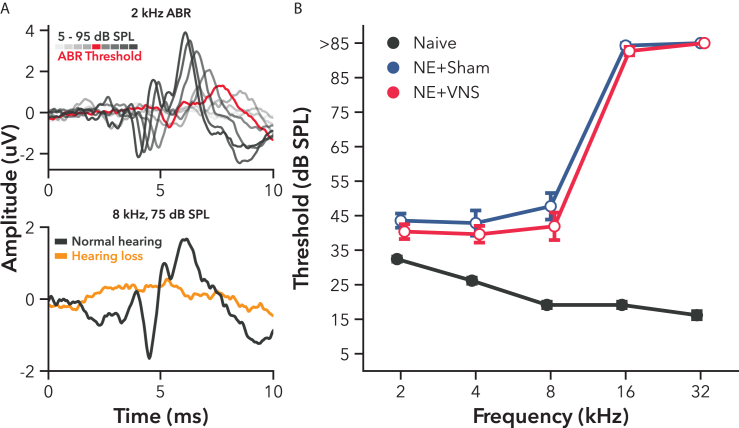
Experiment 1, ABR measurements revealed significant noise-induced hearing loss 3 weeks after noise exposure (A, top) Example ABRs for 2 kHz stimulus over a range of intensities from a noise-exposed rat 3 weeks after exposure. As the intensity of the tone progressively increased from 5 to 95 dB SPL, the amplitude of the mean neural signal likewise increased in amplitude. The threshold, marked in red, indicates the lowest intensity that produced a reliable neural signal. (A, bottom) Example ABRs for 8 kHz, 75 dB SPL stimulus representing a normal neural signal (black) and one after significant hearing loss 3 weeks after noise-exposure (orange). Even at a high stimulus intensity, the hearing loss recording is indistinguishable from background noise. (B) Group ABR thresholds for a set of frequencies across the rat hearing range. One hour of intense, broadband noise exposure led to significantly elevated ABR thresholds compared to non-exposed naive controls at all frequencies tested. Moderate hearing loss was generally observed at 8 kHz and below. Almost all noise-exposed rats had no detectable neural responses to 16 or 32 kHz tones, even at 95 dB SPL. These threshold measurements were used to balance levels of hearing loss between rats randomized into VNS and Sham therapy groups. Open markers indicate post-hoc significance p < 0.05, corrected for multiple comparisons, between NE and naive rats. No differences were present between VNS and Sham groups after randomization. All values are mean ± SEM. Group numbers: naive n = 64, NE + Sham n = 13, NE + VNS n = 14.

After gathering baseline low-frequency tone detection and discrimination thresholds, a randomized subset of rats underwent noise-induced hearing loss (n = 27). Rats were exposed to 1 h of intense broadband noise centered at 22 kHz at 120 dB sound pressure level (SPL), followed by a 3 weeks recovery period. After 3 weeks, auditory brainstem response (ABR) thresholds were recorded across the rat hearing range (Figure 1). NE led to elevated ABR thresholds compared to a set of non-exposed naive controls (naive n = 64, Figure 1B F(2, 88.80) = 476.18, p < 0.001), indicating long-term, significant hearing loss across the entire rat hearing range.^60^ As expected, 16 and 32 kHz tones failed to elicit even minimal neural responses in NE rats at 95 dB SPL tone intensity, compared to mean control thresholds of 19.1 and 16.2 dB SPL, respectively (VNS, 16 and 32 kHz: t(256) = 30.83, p < 0.001, and t(256) = 33.04, p < 0.001. Sham, 16 and 32 kHz: t(256) = 32.48, p < 0.001, and t(256) = 34.07, p < 0.001). Mid-frequency 8 kHz tones still elicited detectable neural responses, but NE led to thresholds increasing by 25.5 dB compared to controls (VNS: t(256) = 9.55, p < 0.001. Sham: t(299) = 11.79, p < 0.001). Thresholds for the lowest tested frequencies of 2 and 4 kHz were also significantly elevated in NE rats, increasing by 9.6 and 15.1 dB, respectively (VNS, 2 and 4 kHz: t(256) = 3.34, p < 0.001, and t(256) = 5.64, p < 0.001. Sham, 2 and 4 kHz: t(256) = 4.82, p < 0.001, and t(256) = 7.21, p < 0.001). NE rats were then randomized into VNS (n = 14) and Sham (n = 13) therapy groups while ensuring their mean ABR thresholds across frequencies remained balanced (VNS vs. Sham, t(88.8) = 1.31, p = 0.39). ABR thresholds across all frequencies remained significantly elevated compared to the naive control set for both VNS (t(87.7) = 22.46, p < 0.001) and Sham (t(89.6) = 24.73, p < 0.001) groups (Figure 1B). VNS and Sham therapy rats then underwent 4 weeks of passive AT pairing VNS with presentation of low-frequency tones.

#### Behavioral hearing thresholds unchanged following noise exposure and passive AT with or without VNS

**Figure 2:**
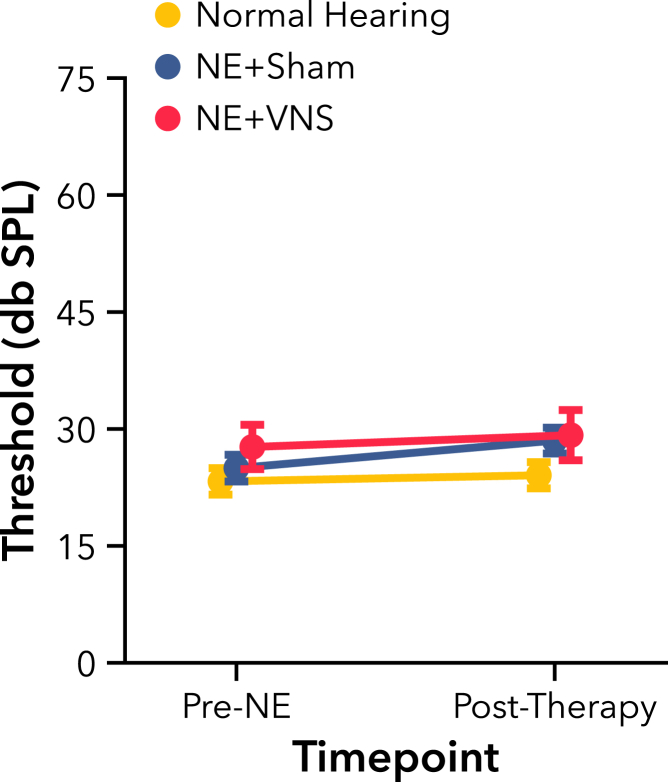
Experiment 1, thresholds for detecting a low-frequency tone were not significantly different between rats with noise-induced hearing loss (NE) and normal hearing controls Receiving 4 weeks of VNS paired with passive AT did not alter behavioral thresholds compared to Sham therapy. All values are mean ± SEM. Group numbers: normal hearing n = 9, NE + Sham n = 13, NE + VNS n = 14.

Following 4 weeks of passive AT either with or without VNS-tone pairing, we re-tested the rats’ tone detection and discrimination thresholds. Given that a compensatory increase in central auditory excitability is commonly observed following hearing loss,^14^^,^^16^ we did not expect the NE groups’ elevated ABR thresholds to be reflected in their behavioral detection thresholds. However, VNS has previously been shown to reduce neural thresholds after passive tone-pairing,^61^ so it was possible rats in the VNS group would achieve lower behavioral thresholds after passive AT. On average, rats in all groups were able to reliably detect tones presented slightly under 30 dB SPL (Figure 2). Overall, low-frequency tone detection thresholds remained unchanged from pre-NE to post-therapy time points (F(1, 35) = 2.00, p = 0.17). NE did not result in elevated detection thresholds compared to normal hearing controls. Additionally, receiving multiple weeks of VNS-tone pairing did not further reduce behavioral thresholds (F(2, 33) = 1.37, p = 0.27).

**Figure 3:**
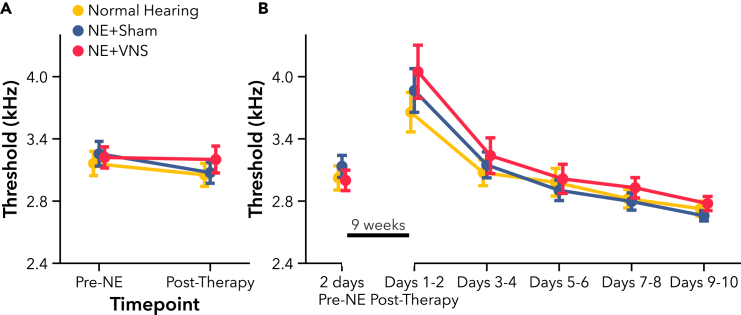
Experiment 1, discrimination thresholds for low-frequency tones were unaffected by broadband noise exposure (A) Thresholds for discriminating a low-frequency tone from higher-frequency tones were not significantly different between rats with noise-induced hearing loss (NE) and normal hearing controls. Receiving 4 weeks of VNS paired with passive acoustic therapy did not alter behavioral thresholds compared to Sham therapy. (B) All rats had elevated thresholds after a 9 weeks break from behavior. No significant difference was seen between NE rats and normal hearing controls. All values are mean ± SEM. Group numbers: normal hearing n = 9, NE + Sham n = 13, NE + VNS n = 14.

After detection threshold testing, we evaluated the effect of AT on frequency discrimination thresholds. Rats were required to respond to the 2 kHz target tone, and withhold responding to higher frequency tones. Non-target frequencies were chosen using an online adaptive algorithm, with most trials presenting frequencies close to individual rats’ discrimination thresholds (see STAR Methods and Figure S3). Rats were able to consistently discriminate the 2 kHz target tone from higher frequency tones, reliably withholding from responding to non-target tones half an octave or more away (Figure 3A). Mean low-frequency tone discrimination threshold estimates decreased very slightly from pre-NE to post-therapy time points (F(1, 35) = 4.44, p < 0.05), from approximately 3.2 to 3.1 kHz. Unexpectedly, hearing loss did not result in elevated discrimination thresholds compared to normal hearing controls (F(2, 33) = 0.21, p = 0.82).

Our discrimination threshold estimates were measured over 10 days to ensure stability. Previous work using a similar plasticity-inducing passive stimulation protocol demonstrated transient enhancements in behavioral discrimination following treatment in healthy rats.^62^ To determine if NE or multiple weeks of VNS paired with passive AT drove any transient differences in discrimination, we examined the change in threshold estimation for each group over time. All rats had elevated thresholds on the first days of testing after a 9 weeks intermediary period of no discrimination behavior, only reliably discriminating non-target tones a full octave away from 2 kHz (Figure 3B F(1, 35) = 57.46, p < 0.001). However, neither NE nor 4 weeks of passive AT paired with VNS significantly altered these initial post-NE threshold estimates compared with normal hearing controls (F(2, 33) = 0.36, p = 0.70). Discrimination thresholds rapidly returned to baseline levels and continued to decrease down to 2.8 kHz over further testing days (F(1, 141) = 171.93, p < 0.001), indicating an auditory benefit from continued training, even with low- and mid-frequency hearing loss. However, no transient differences ever emerged between groups (F(2, 173.88) = 1.49, p = 0.23). These results demonstrate overall stability of simple tonal detection and discrimination even after significant noise-induced hearing loss. No behavioral deficit was observed, and VNS did not improve rats’ behavioral performance.

#### VNS paired with active AT fails to improve learning or performance on speech discrimination tasks

**Figure 4:**
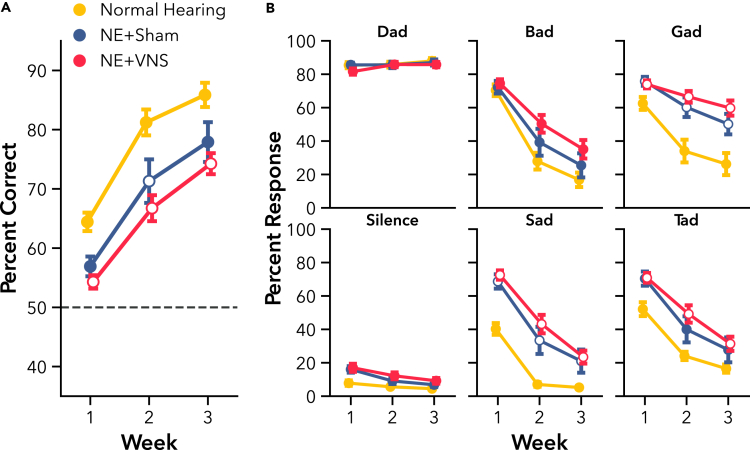
Experiment 1, VNS paired with active AT did not improve speech discrimination learning rate or overall performance after hearing loss (A) Compared to normal hearing rats, hearing loss rats (NE) showed significant impairment on speech discrimination over 3 weeks of training. No significant differences were observed between NE + VNS and NE + Sham performance. NE rats showed differential impairments depending on the presented speech sound. (B) All rats reliably responded to the target sound, “Dad,” and withheld responding to silent catch trials. Hearing loss significantly impaired correct rejection of the sounds “Gad,” “Sad,” and “Tad” compared to normal hearing controls. No significant differences were observed between NE + VNS and NE + Sham response rates for any sound. Open markers indicate post-hoc significance p < 0.05, corrected for multiple comparisons, between NE and normal hearing rats. Dashed gray line represents chance performance. All values are mean ± SEM. Group numbers: normal hearing n = 9, NE + Sham n = 13, NE + VNS n = 14.

We next sought to determine if NE impaired learning and performance on a novel speech discrimination task, and whether VNS paired with active AT could improve behavioral outcomes. Rats trained on a novel Go/No-go speech discrimination task for 3 weeks, learning to respond to the target speech sound, “Dad,” and withhold responding to non-target sounds, “Bad,” “Gad,” “Sad,” and “Tad.” Subjects in the VNS group received VNS paired with correct hits to “Dad” during training sessions, while Sham rats received training only. All groups improved their discrimination ability over weeks of active AT, performing well-above chance levels (Figure 4A F(1, 69) = 404.33, p < 0.001). There was a significant difference in performance between the groups over the 3 weeks training period (F(2, 73.09) = 3.28, p < 0.05), but no interaction of group and time (F(2, 69) = 0.18, p = 0.83), indicating that hearing loss impaired performance without affecting overall learning rate. NE rats performed significantly worse than normal hearing controls (t(33) = 3.44, p < 0.01). However, there was not a significant difference between VNS and Sham groups (t(33) = 1.21, p = 0.41), indicating that pairing VNS with active AT did not further improve overall discrimination performance compared to training alone.

We tested the impact of NE on learning for the individual sounds used in the task. As rats learned the task, their rate of correctly responding to the target sound, “Dad,” increased while their rate of responding to the non-targets predictably decreased (Figure 4B). Since each speech sound elicits distinct spectrotemporal neural activity,^63^ hearing loss would be expected to differentially impair discrimination of some sounds more than others. As expected, all rats showed improved response rates over time, correctly responding to the target or withholding from responding to non-targets (F(1, 589) = 549.64, p < 0.001). As with overall performance, NE and hearing loss significantly impaired response rates (F(2, 63.77) = 3.79, p < 0.05), but did not alter rate of improvement over time (F(2, 589) = 1.30, p = 0.27). We observed a differential effect of hearing loss on sound as expected, indicating that some speech sounds were more difficult for NE rats to discriminate than others (F(10, 589) = 9.27, p < 0.001). Post-hoc testing showed significant impairments for discriminating the sounds, “Gad” (t(58) = 4.99, p < 0.001), “Sad” (t(58) = 5.57, p < 0.001), and “Tad” (t(58) = 3.69, p < 0.01), but not “Bad” (t(58) = 2.36, p = 0.15) or the target sound, “Dad” (t(58) = 0.24, p = 1.00). Response rates were also not different for silent catch trials (t(58) = 1.22, p = 0.80). Additional post-hoc testing did not show any differences in response rates between VNS and Sham groups, confirming that adjunctive VNS treatment did not facilitate additional recovery of auditory function during active AT.

**Figure 5:**
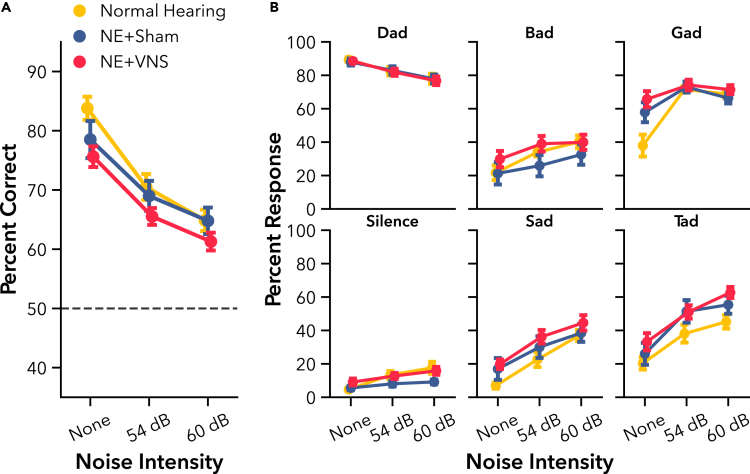
Experiment 1, VNS did not improve speech-in-noise discrimination performance after hearing loss (A) The addition of competing speech-shaped background noise decreased speech discrimination performance in all rats. Unexpectedly, increasing background noise did not lead to an increased deficit in performance in rats with hearing loss (NE) compared to normal hearing controls. This remained true even when examining response rates for each individual sound. (B) Although the addition of noise impacted perceptual acuity for speech sounds differentially, no significant differences in response rates were observed between NE rats and normal hearing controls. Dashed gray line represents chance performance. All values are mean ± SEM. Group numbers: normal hearing n = 9, NE + Sham n = 13, NE + VNS n = 14.

One of the most common issues following hearing loss is difficulty understanding speech embedded in competing background noise. Following speech discrimination training in quiet, we tested the impact of adding two different levels of speech-shaped background noise on task performance. As noise intensity increased, overall discrimination performance dropped, as expected (Figure 5A F(2, 66) = 200.11, p < 0.001).^64^ Surprisingly, hearing loss did not lead to increased impairment in noise compared to normal hearing controls (F(2, 33) = 1.88, p = 0.17).^5^^,^^10^^,^^65^ We examined performance by response rates to the various speech sounds in different background noise levels to determine if NE led to any differential spectrotemporal impairments in noise (Figure 5B). Adding noise impaired response rates for all sounds (F(2, 581) = 74.68, p < 0.001), but again no overall difference was seen between groups (F(2, 33) = 1.65, p = 0.21). As in quiet, there was a significant interaction of sound and group on response rates while discriminating in noise (F(10, 581) = 3.63, p < 0.001). However, further post-hoc testing did not show any significant differences between normal hearing and NE rats, or between VNS and Sham groups, for any individual speech sound. Additionally, consistent with our prior findings, VNS paired with active AT did not further enhance speech-in-noise performance compared to equivalent AT with sham stimulation.

### Experiment 2

In Experiment 1, all NE rats were ultimately able to successfully perform the tasks regardless of treatment. By comparison, Sham groups in stroke models that most benefit from VNS-aided rehabilitation often struggle to complete trials even after weeks of training.^34^ It is possible that the deficit induced after noise-exposure was too small to realize potential benefits from VNS paired with AT, or that the tasks were too easy. In Experiment 2, we paired VNS in passive AT with speech sounds over a range of intensities and tested NE rats on a more difficult fricative speech discrimination task.^63^ We additionally varied NE parameters to produce a group of rats with a more profound hearing loss deficit.

#### Noise exposure causes severe to profound hearing loss

After reaching proficiency on the basic Go/No-go procedure, rats underwent noise-induced hearing loss (n = 36). The frequency and intensity parameters of the broadband noise used in this experiment varied from a center frequency of 4–22 kHz and an intensity of 120 or 125 dB SPL. These parameter choices can produce a mixture of hearing loss outcomes, including profound hearing loss across the entire rat hearing range.^60^

**Figure 6:**
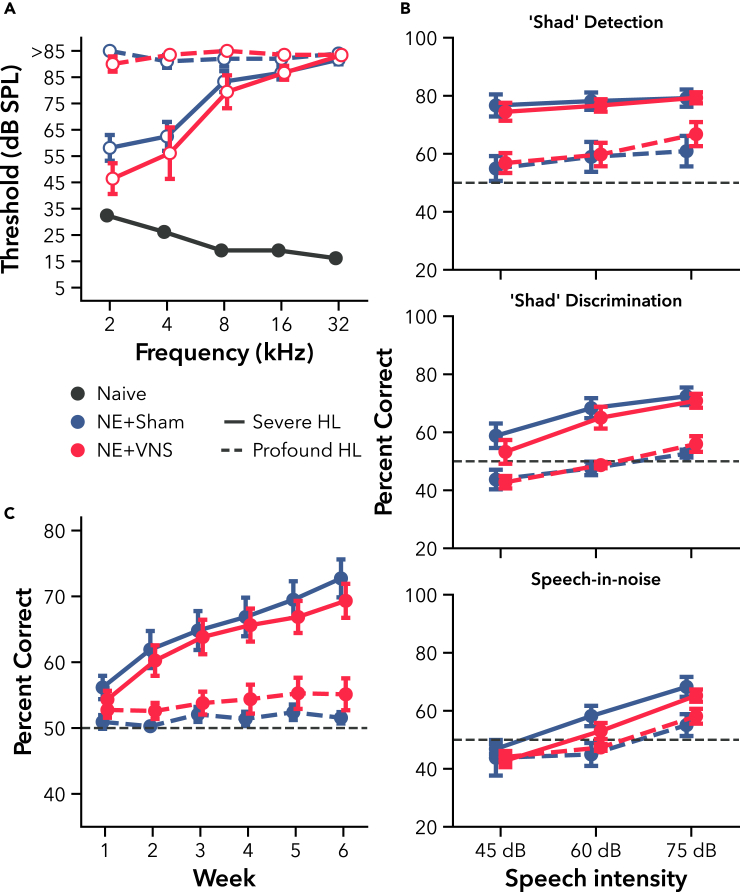
Experiment 2, VNS paired with speech during passive AT did not improve speech detection, discrimination, or SIN performance after hearing loss (A) NE rats were categorized as having “severe” (solid line) or “profound” (dashed line) hearing loss (HL) based on their ABR thresholds 4 weeks after noise exposure. Profound HL rats were characterized by near deafness across the entire rat hearing range, while Severe HL rats suffered similar high frequency deficits but had only moderate shifts in low-mid frequency thresholds compared to non-exposed naive controls. Open markers indicate post-hoc significance p < 0.05, corrected for multiple comparisons, between NE and naive rats. No differences were present between VNS and Sham groups within their HL categories after randomization. (B) Passive AT pairing VNS with presentation of the speech sound “Shad” at a variety of intensities did not improve performance for “Shad” detection (top), discrimination from competing sounds “Had,” “Jad,” and “Sad” (middle), or discrimination in 60 dB SPL speech-shaped background noise (bottom) compared to Sham for either severe or profound HL. (C) Rats with severe HL improved on “Shad” discrimination over 6 weeks of training, but VNS during passive AT did not lead to faster learning or better performance compared to Sham. Rats with profound HL failed to significantly improve in discrimination regardless. Dashed gray line represents chance performance. All values are mean ± SEM. Group numbers (except for speech-in-noise, B, bottom): naive n = 64, NE + Sham/Severe HL n = 12, NE + VNS/Severe HL n = 9, NE + Sham/Profound HL n = 5, NE + VNS/Profound HL n = 10. Group numbers for speech-in-noise: NE + Sham/Severe HL n = 11, NE + VNS/Severe HL n = 8, NE + Sham/Profound HL n = 3, NE + VNS/Profound HL n = 8.

As in Experiment 1, NE increased ABR thresholds compared to a set of non-exposed naive controls across all tested frequencies (naive n = 64, Figure 6A F(4, 104.74) = 491.05, p < 0.001). Rats with thresholds over 80 dB SPL across all tested frequencies were considered to have Profound hearing loss (n = 15), while rats with any intact low- or mid-frequency hearing were considered to have Severe hearing loss (n = 21). Both Profound (t(109.5) = 34.89, p < 0.001) and Severe (t(96.2) = 31.69, p < 0.001) hearing loss groups remained significantly different from non-exposed controls after sorting the rats by their ABR deficits. Rats were then randomized within each hearing loss category into VNS (Severe n = 9; Profound n = 10) and Sham (Severe n = 12; Profound n = 5) therapy groups while ensuring their mean ABR thresholds across frequencies remained balanced (Severe VNS vs. Sham t(97.2) = 1.28, p = 0.61, Profound VNS vs. Sham t(113.0) = 0.14, p = 1.00). Thresholds remained significantly elevated compared to the naive control set for all groups (Figure 6A). Rats in the VNS therapy groups then underwent 4 weeks of passive AT pairing VNS with presentations of the target speech sound, “Shad.”

#### Passive AT pairing VNS with fricative speech fails to improve performance on multiple speech tasks

Following 4 weeks of passive AT either with or without VNS-speech pairing, we tested rats on speech detection and a novel discrimination task, including speech-in-noise. For detection, the speech sound “Shad” was pseudorandomly presented at either 45, 60, or 75 dB SPL. As expected, rats generally performed better at higher presentation intensities (Figure 6B, top, F(2, 68) = 15.69, p < 0.001). Although rats with profound hearing loss were able to detect speech presentation above chance levels, they were significantly worse than rats with severe hearing loss (F(1, 32) = 25.14, p < 0.001). Similar to the Experiment 1 tone detection results, multiple weeks of passive AT with VNS did not enhance speech detection abilities in either hearing loss group compared to Sham (F(1, 32) = 0.06, p = 0.81). These results suggest that VNS therapies do not generally facilitate improvements in auditory perceptual thresholds after hearing loss.

After detection testing, rats were trained to discriminate the target speech sound, “Shad,” from non-target sounds, “Had,” “Jad,” and “Sad” over a period of 6 weeks (Figure 6C). All speech sounds were pseudorandomly presented at 45, 60, or 75 dB SPL. Discrimination improved through weeks of training (F(1, 171.00) = 153.21, p < 0.001), but only for rats categorized with severe hearing loss. Performance differences between hearing loss groups became more substantial over time (F(1, 171.10) = 79.64, p < 0.001). Although rats with profound hearing loss were able to perform better than chance, post-hoc testing indicated that there was no significant difference between their week 1 and week 6 performances (t(172) = 2.03, p = 0.09). Consistent with the speech discrimination results from Experiment 1, the VNS treatment groups for both severe and profound hearing loss failed to perform better or learn faster than Sham (F(1, 42.01) = 0.02, p = 0.88).

We examined discrimination ability for the three different intensities of speech sounds averaged over the last week of training (Figure 6B, middle). As expected, there was a significant difference between hearing loss groups (F(1, 32) = 28.67, p < 0.001), and rats in all groups performed better at higher intensities (F(2, 68) = 48.19, p < 0.001). There was no interaction of hearing loss and speech intensity (F(2, 68) = 1.89, p = 0.16), indicating that although profound hearing loss substantially impaired rats’ ability to discriminate speech compared to severe hearing loss, increasing the intensity of the sounds helped in roughly equal amounts for each group. Previously receiving weeks of passive AT pairing speech at multiple intensities with VNS failed to differentiate discrimination performance at any tested speech intensity compared to Sham (F(1, 32) = 0.14, p = 0.71).

Rats then performed speech discrimination with the addition of 60 dB SPL speech-shaped background noise. Six rats did not finish this final task and were removed from analysis (speech-in-noise group numbers: VNS/Severe n = 8, VNS/Profound n = 8, Sham/Severe n = 11, and Sham/Profound n = 3). The addition of 60 dB SPL speech-shaped background noise impaired discrimination performance for rats with severe hearing loss (Figure 6B, bottom, F(1, 161.61) = 28.13, p < 0.001). Rats with severe hearing loss still performed significantly better in noise than rats with profound hearing loss (t(44.7) = 2.05, p < 0.05), though not for 45 dB sounds presented at a −15 dB signal-to-noise ratio (t(62.1) = 0.51, p = 0.87). VNS treatments once again failed to provide any benefit to discrimination in noise for either hearing loss group (F(1, 26) = 0.18, p = 0.68). Post-behavior ABRs were then recorded in a subset of rats and within-subject changes in ABR thresholds calculated (Figure S6). No significant differences were found between VNS and Sham treatment groups, suggesting that passive AT with VNS also failed to generate peripheral recovery. Altogether, the results of both Experiment 1 and 2 demonstrate that neither passive nor active AT in this noise-induced hearing loss model, utilizing tones or speech sounds across a range of stimulus intensities, benefit from adjunctive VNS treatment.

## Discussion

In this study, we attempted to improve auditory behavior in a noise-induced hearing loss model through rehabilitation paired with VNS. In the first experiment, rats had significantly elevated ABR thresholds across their hearing range several weeks after noise-exposure, but did not demonstrate a similar shift in behavioral thresholds for tone detection or frequency discrimination. However, hearing loss impaired their performance on a novel speech discrimination task. All groups improved on the task over time, though significant deficits still existed compared to normal hearing controls after several weeks of training. The addition of speech-shaped background noise disrupted performance in all groups, but hearing loss did not result in further deficits. VNS paired with either passive or active AT failed to drive recovery of perceptual ability for speech discrimination compared to Sham controls. A second experiment tested pairing passive AT with speech in rats with either severe or profound hearing loss. We again found that pairing VNS with passive AT after hearing loss failed to improve performance on multiple speech tasks compared to Sham controls. VNS failed to improve performance in rats with moderate to profound hearing loss for any of the behavioral tasks tested, including multiple speech discrimination tasks.

We first examined the impact of severe hearing loss on tonal perceptual acuity, and whether multiple weeks of passive AT pairing VNS with a low-frequency tone could improve behavioral performance. Intense, broadband NE elevated ABR thresholds across the rat hearing range (Figure 1B). Behavioral thresholds for detecting presentation of a low-frequency tone, however, were unaltered in rats with hearing loss compared to normal hearing controls (Figure 2). This result likely reflects an increase in central gain, a typical neurophysiological adaptation in the brain following traumatic hearing loss. Decreased inhibition and elevated excitability in auditory networks compensate for the reduction in afferent cochlear input to maintain spike rate, permitting continued detection of relatively quiet sounds, though potentially at the expense of other auditory processes.^14^^,^^16^^,^^17^

Intense NE also did not induce a low tone frequency discrimination deficit (Figure 3). Hearing loss is known to produce significant shifts in tonotopic organization, from cochlea to cortex,^12^^,^^15^^,^^41^^,^^66^^,^^67^ and has been previously connected with reductions in frequency resolution.^15^^,^^65^^,^^68^^,^^69^^,^^70^^,^^71^^,^^72^ Alternatively, some particular cases of hearing loss may actually produce a specific local improvement in discrimination^73^ or else have no effect at all.^70^^,^^71^ It is possible that the hearing loss model used in Experiment 1 may not generate significant deficits in pure tone frequency discrimination within the intact range of hearing. The task design may also have been too simple to enable observation of a frequency deficit.

Our use of a 2 kHz tone for behavioral testing and passive AT in Experiment 1 may have been sub-optimal, potentially being too low frequency within the rat hearing range to reveal or treat any tonal hearing loss deficits following NE. The 2 kHz frequency was selected over higher frequency alternatives due to the expectation of high variance in hearing loss outcomes that can be typical of NE models (see Figure S7, 8 kHz stimulus), as well as the expected limits of rat frequency discrimination. A similar training procedure used by Reed et al. resulted in rats only reliably discriminating tones 0.5 to 1 octave away from the target frequency.^62^ Use of a higher frequency target tone in our experiments, such as 6 kHz, could have resulted in many of the non-target tones near rats’ discrimination thresholds having frequencies that noise-exposed subjects may not have been able to clearly hear (e.g., 9–12 kHz), thus confounding the measurement entirely. Using the lower frequency 2 kHz target allowed us to reliably measure detection and discrimination thresholds across all subjects while still demonstrating a significant deficit in ABR thresholds after NE. However, future work may benefit from exploring similar tasks and treatments as tested here but instead utilizing highly individualized stimulus selections and training strategies to mitigate this concern.

Hearing loss is frequently associated with struggles to understand spectrotemporally modulated sounds, like speech. Although hearing aids can provide a benefit to some users, they may also make speech more difficult to understand, particularly in noise.^5^^,^^7^^,^^8^ Intense, active AT may help alleviate these deficits, enabling individuals to participate in every day conversation more easily and with greater clarity.^19^^,^^25^ In Experiment 1, we delivered VNS to rats with hearing loss during active AT on a novel speech discrimination task, a rehabilitation strategy that has consistently amplified long-term behavioral recovery in motor deficit models and in clinical populations compared to training without VNS.^33^^,^^34^^,^^51^ Rats with hearing loss had significant performance deficits compared to normal hearing rats, but continuously improved with training (Figure 4). However, VNS paired with correct responses to the target sound did not accelerate improvement or increase speech discrimination ability above Sham control rats. Previous studies in motor deficit models have reported an association between enlarged representations of motor movements in cortex after similarly pairing VNS with motor rehabilitation and a substantial recovery of motor function.^33^^,^^34^^,^^38^ The lack of auditory improvement in the VNS group in this study may highlight key differences in either plasticity mechanisms or the manifestation of behavior between the auditory and motor systems. However, VNS paired with rehabilitation has also been reported to improve somatosensory thresholds and behavior following maladaptive changes in the central nervous system after injury.^33^^,^^36^^,^^47^ The type of injury, then, may be the primary factor determining if VNS can improve recovery of function.

The addition of speech-shaped background noise decreased speech discrimination performance for all rats, as expected (Figure 5). Surprisingly, we did not see a significant difference in perceptual impairment between rats with and without hearing loss. Difficulty comprehending speech in noise is one of the most frequent issues encountered in those with hearing loss,^5^^,^^10^^,^^65^ even when listening in quiet conditions is otherwise unimpaired.^74^ It is possible that we did not observe an expanded discrimination deficit in rats with hearing loss due to the prior 3 weeks of active AT, potentially demonstrating a considerable benefit of active training by increasing perceptual acuity, even in challenging conditions.^75^ However, our experimental design lacked the necessary controls to verify this specific outcome. Alternatively, this particular model of hearing loss may not generate a sizable speech-in-noise deficit, even when speech discrimination in quiet is degraded. Regardless, receiving multiple weeks of VNS paired with active AT did not differentiate VNS and Sham performances in the speech-in-noise discrimination task. Hearing loss thus stands out as a condition that may substantially benefit from active rehabilitation, but, unlike other neural deficit models, did not show an adjunctive training benefit when paired with VNS.

It was possible that many of the design choices in Experiment 1 could mask a potential benefit of VNS paired with AT under alternative conditions. Rats had relatively intact low- to mid-frequency hearing, passive AT paired VNS with simple tones, and the speech discrimination task used consonant sounds known to evoke strong, bursting activity across rat auditory cortex.^63^ We performed a second experiment that included rats with profound hearing loss across the entire rat hearing range (Figure 6A), paired VNS with speech sounds presented over a range of intensities, and tested rats’ ability to discriminate fricative or affricate consonant sounds known to more weakly activate mid-frequency regions of auditory cortex.^63^ Rats with profound hearing loss struggled to report simple detection of speech sounds (Figure 6B, top), and failed to improve on discrimination over 6 weeks of training (Figure 6B, middle; Figure 6C), regardless of VNS or Sham therapy treatment. While rats classified as having only severe hearing loss were able to detect speech sounds and improve on discrimination over time, VNS paired with speech during passive AT failed to enhance learning or performance, including in noise (Figure 6). VNS treatment also failed to drive significant recovery of within-subject ABR thresholds (Figure S6). Taken together, the results from our experiments demonstrate that VNS rehabilitation strategies which have been found to enhance recovery in motor and somatosensory deficit models do not similarly improve functional outcomes when paired with auditory rehabilitation after noise-induced hearing loss. Novel auditory rehabilitation strategies should be pursued to facilitate optimal recovery of function where possible.

Although VNS paired with passive and active AT failed to improve auditory behavior after noise-induced hearing loss in this study, it remains possible that alternative pairing strategies could yield benefits. Whitton and colleagues reported significant improvements in patients with hearing loss using gameified, closed-loop AT with varying amounts of background noise.^19^ Integrating VNS with a closed-loop training system would permit testing treatment strategies such as stimulating only during optimal performance, which may augment reinforcement signals to promote continued utilization of optimal patterns of neural activity.^56^ Whitton also reported that the benefits they observed did not continue once training stopped. The benefits from pairing VNS with rehabilitation are consistently long-lasting, even after treatment ends.^34^^,^^36^ The use of VNS during training may thus facilitate maintenance of long-lasting recovery after treatment stops, even if it does not provide an overall improvement in performance during the training period. Alternatively, repeatedly pairing VNS with training stimuli immediately prior to AT sessions may accelerate training and improve performance through enhanced engagement of neuromodulatory centers like the noradrenergic locus coeruleus (LC).^57^^,^^76^^,^^77^ Other alternative strategies include attempting to improve cognition and verbal working memory^78^^,^^79^^,^^80^^,^^81^^,^^82^^,^^83^^,^^84^ to potentially make following conversations easier,^3^^,^^5^^,^^8^^,^^85^^,^^86^ testing VNS with hearing loss resulting from aging rather than traumatic insult,^44^ or pairing VNS with a rich variety of sounds to try to generalize recovery beyond simple training stimuli.^34^^,^^35^ It is also possible that VNS may be able influence recovery if delivered acutely after an injury-inducing event, prior to the occurrence of innate neurophysiological processes such as central gain.^87^

Improvements in audition for patients with hearing loss must derive from both a restoration of peripheral input signals and plasticity of the downstream auditory processing networks.^13^ Hearing aids may provide peripheral benefit, but this is not enough. Utilizing AT therapies to drive beneficial plasticity in dysfunctional auditory circuits is necessary for generating optimal recovery.^13^^,^^21^^,^^25^^,^^88^ Beneficial central plasticity is facilitated through engagement of neuromodulatory networks that control attention and arousal, like the LC. A recent study in a rat model for cochlear implant after hearing loss demonstrated that central plasticity and LC activity after implant was predictive of functional outcome and behavioral improvement.^77^ Stimulation of the LC prior to AT led to enhanced learning and performance, suggesting that optimal behavioral adaptation to alterations in auditory input may require adjunctive treatments that engage neuromodulatory systems. VNS efficiently drives phasic LC activity,^57^ and has consistently facilitated auditory plasticity in healthy subjects.^41^^,^^61^^,^^89^ This study is the first attempt to facilitate the necessary plasticity to improve auditory perceptual acuity through an adjunctive VNS therapy paired with rehabilitation after moderate to profound hearing loss, a strategy that has otherwise generated significant improvements for recovery across a wide range of sensory and motor disorders in both rodent models and in clinical populations. Future VNS-aided therapy designs may yet yield highly beneficial clinical treatment outcomes for hearing loss through targeted manipulation of neuromodulatory activity vital to the generation of central plasticity.

A key distinction between this study and all previous studies that explore VNS paired with rehabilitation is the significant damage done to peripheral receptors. The most documented rehabilitation benefit from VNS treatment is enhanced recovery following stroke, which is strictly central in pathology. Potential benefits from facilitating central plasticity through VNS-paired rehabilitation may thus be limited by a ceiling effect due to the destruction of peripheral inputs. An exciting area of research has been investigating regeneration of the auditory periphery after damage.^90^^,^^91^^,^^92^^,^^93^ Restoration of peripheral input could expand the degree of recovery possible, though it is likely that interpretation of the recovered signals would require significant adaptation by auditory circuits in the brain.^77^^,^^88^ We have previously shown that VNS improves motor and sensory recovery following peripheral nerve injury by facilitating circuit adaptation in the brain after peripheral axon regeneration.^33^ Because the peripheral receptors in that study were intact, VNS paired with rehabilitation was able to fully engage the nervous system and adapt neural circuits to the regenerated signals. Should restoration of the auditory periphery prove clinically feasible, patients would likely substantially benefit from adjunctive VNS-paired auditory therapies aimed at increasing the adaptability of auditory circuits to the regenerated input signals.

As the number of people suffering from hearing loss continues to grow, it is vital that research on auditory rehabilitation strategies continue to develop new ideas and therapies. AT to alleviate deficits associated with hearing loss has been a subject of increasing interest for over 20 years, yet little improvement has been made in the field. It is clear now that hearing loss is a multifaceted problem extending beyond a simple loss of peripheral sensory input. Future research should continue to focus efforts on understanding which aspects of central plasticity after hearing loss are maladaptive, which are necessary for long-term improvements, and seek novel ways to mitigate both auditory and cognitive deficits. Additionally, more detailed examination of the peripheral damage from noise-induced hearing loss and the potential for recovery following VNS may help reveal limits of rehabilitative treatments without prior restoration of peripheral signals. Nuanced neurophysiological investigations of measures such as speech ABRs and cortical activity before and after VNS may add to our growing understanding of central and peripheral changes after hearing loss that lead to behavioral deficits. VNS treatments are known to follow an inverted-U dose-response curve, failing to drive plasticity when stimulation intensity is too low or too high.^94^^,^^95^ More work is necessary to determine the optimal VNS parameter settings necessary to drive therapeutic recovery, which may vary within subject and with condition.

### Limitations of the study

Our study focused on testing the viability of improving auditory perception after hearing loss by using VNS treatment strategies that are consistently effective in other VNS-based rehabilitation and neuroplasticity domains. Due to this limited scope, we did not explore granular physiological changes to peripheral receptors in the cochlea or in auditory circuits beyond recording tonal ABRs (Figures 1 and 6). Similar experiments in the future may be benefit from investigating subject-specific trauma and adaptations that could clarify when VNS may or may not be an effective therapeutic, or help reveal novel strategies for tailoring treatment to specific problems. For example, our use of a low-frequency target tone in Experiment 1 was partially motivated by the need to control against a high degree of inter-subject variability in trauma outcome. Additionally, while we tested a broad range of hearing loss across experiments, from moderate to nearly deaf, the current study was limited to a single noise trauma model. The use of other translational models for hearing loss, particularly with aged subjects, is a compelling avenue of additional research for adjunctive neuromodulatory therapies like VNS. Our VNS parameters were also fixed to a single set. While the parameters chosen are known to produce robust plasticity and behavioral recovery across cortical areas and in a variety of injuries,^36^^,^^38^^,^^40^^,^^41^^,^^61^^,^^96^^,^^97^ this is the first VNS study testing rehabilitation after severe injury to a cranial nerve, and it cannot be ruled out that alternative parameter choices may have been effective. Impedance was monitored daily to ensure closed and functional connection to stimulators during treatments, but these measurements were not recorded for offline analysis, and so we cannot report on possible trends over time that may have influenced efficacy of stimulation. VNS was also paired with active AT in Experiment 1 seven weeks post-implant recovery, which is a slightly longer treatment timeline than many similar, previous VNS experiments, and may have been a limiting factor in driving recovery. However, all subjects included in our analyses were determined to still have a functional cuff at the end of the experiments through activation of the Hering-Breuer reflex under anesthesia (see STAR Methods). Finally, although we have not previously found sex to be a determining factor in VNS-based treatment outcomes, the current study only utilized female rats, and we cannot rule out the possibility of a sex-specific result.

## STAR★Methods

### Key resources table

**Table:** 

REAGENT or RESOURCE	SOURCE	IDENTIFIER
**Software and algorithms**		

Python version 3.11	Python Software Foundation	https://www.python.org/
MATLAB 2021b	MathWorks	https://www.mathworks.com/
R version 4.1.1	R Foundation	https://www.r-project.org/
BioSigRZ	Tucker-Davis Technologies	https://www.tdt.com/component/biosigrz-abr-dpoae-software/

### Resource availability

#### Lead contact

Further information and requests for resources should be directed to and will be fulfilled by the lead contact, Alan Carroll (alan.carroll@utdallas.edu)

#### Materials availability

This study did not generate new unique reagents.

#### Data and code availability

• All data reported in this paper will be shared by the lead contact upon request.
• All code utilized in this paper will be shared by the lead contact upon request.
• Any additional information required to reanalyze the data reported in this paper is available from the lead contact upon request.

### Experimental model and study participant details

A total of 101 adult and age-matched (4–12 months old) female Sprague-Dawley rats (Charles River Laboratories) were used for this study. Of these, 29 were excluded from final analysis due to VNS cuff or connector failure or surgical death. In Experiment 1, 50 rats were used and 14 excluded from analysis. Rats were randomized into Normal Hearing (n = 9) or hearing loss (NE + VNS/Sham, n = 27) groups following initial behavioral shaping. NE rats were further randomized into VNS (n = 14) and Sham (n = 13) therapy groups after undergoing noise-induced hearing loss and subsequent measurement of tonal ABR thresholds (Figure 1). In Experiment 2, 51 rats were used and 15 excluded from all analysis Rats were categorized into Severe or Profound hearing loss groups based on shifts in ABR thresholds (Figure 6A). Within each hearing loss category, they were randomized into VNS (Severe n = 9, Profound n = 10) and Sham (Severe n = 12, Profound n = 5) therapy groups. An additional 6 rats were excluded from speech-in-noise testing due to loss of VNS skull connectors. Group numbers for speech-in-noise analysis were: VNS/Severe n = 8, VNS/Profound n = 8, Sham/Severe n = 11, and Sham/Profound n = 3.

Rats were housed on a 12:12 reversed light cycle environment. All behavior and VNS therapy sessions were performed during the dark cycle to increase daytime activity. Rats were habituated to researcher handling and exposed to reward sugar pellets (45 mg dustless precision pellet, BioServ, Frenchtown, NJ) for 1 week before beginning the experiment. During behavioral training, rats were food deprived to no less than 85% of their initial body weight and were supplemented daily and over weekends to maintain a healthy weight. Water was available *ad libitum*. All handling, housing, surgical, and behavioral training were approved by the University of Texas at Dallas Institutional Animal Care and Use Committee. All experiments were performed in accordance with relevant guidelines and regulations. All experiments described here conform to the ARRIVE guidelines.

### Method details

#### Behavior

All tasks used an auditory operant Go/No-go procedure requiring rats to respond to target sounds and withhold responding to non-target sounds, as in previous studies.^60^^,^^62^^,^^63^^,^^67^^,^^98^ Training was performed in acoustically transparent cages inside of double-walled, sound-attenuated booths remotely monitored by webcams and controlled by custom Python or MATLAB programs. All behavior sessions were 60 min long, and rats trained twice per day, 5 days per week, with a minimum 90 min break between sessions. Trials were presented every 8 s. Rats typically performed about 300 trials per session, regardless of behavior task. Performance on the Go/No-go tasks was quantified as the mean percentage of correct responses to target, non-target, and silent catch trials. Rats were initially shaped to use the operant training booth by nosepoking through an IR break-beam sensor within 3 s of presentation of a target tone (Experiment 1: 500 ms, 2 kHz, 60 dB SPL) or the speech sound "Shad" (Experiment 2).^60^^,^^63^ Successful responses triggered delivery of a reward pellet. Responding during silent catch trials, or to non-target sounds in later stages, resulted in an overhead house light turning off and an 8 s timeout. Rats were considered proficient in the basic Go/No-go procedure after reaching a performance criterion of 10 cumulative sessions with at least 75% correct responses to the target sound vs. silent catch trials.

#### Experiment 1

##### Pre-training

After successfully training on basic Go/No-go detection of a 2 kHz tone, rats trained to discriminate the 2 kHz target tone from an 11.3 kHz non-target tone. This stage was used only to ensure that rats were capable of performing simple discriminations, and therefore rats were only required to achieve a single session with at least 75% correct discrimination performance. Rats were then randomized into Normal Hearing (n = 9) and hearing loss (NE + VNS/Sham, n = 27) groups while ensuring their mean training time to Go/No-go proficiency was balanced between the groups. After training rats to use the Go/No-go procedure to detect and discriminate the 2 kHz target tone, we estimated their thresholds for frequency discrimination and tone detection by fitting psychometric functions using the psi-marginal adaptive algorithm.^99^ This method selects stimuli to adaptively reduce the uncertainty of the threshold measurement for a psychometric function while allowing flexibility in modeling the asymptotic "lapse" and "guess" rates of the fitted curve. The guess rate is the minimum rate of responding or withholding from responding in a task, when a subject can be assumed to simply be guessing during any given trial. The lapse rate is the maximum rate of responding or withholding from responding in a task, when a subject is presumed to only behave incorrectly due to a lapse in attention. These rates can be quite variable within and between rats performing these tasks. The psi-marginal sampling method was specifically designed to allow dynamic lapse/guess rates while robustly estimating psychometric thresholds.

##### Frequency discrimination testing

Rats had to discriminate the 2 kHz target tone from 30 potential higher frequency semitones using 1/12 octave steps, ranging from 2.1 to 11.3 kHz (all tones were 500 ms and 60 dB SPL). The target tone, or one of the possible non-target tones, each had a 45% chance of being pseudorandomly presented on every trial. The remaining 10% of trials were silent catch trials. The frequency of a non-target tone was chosen adaptively using the psi-marginal algorithm.^99^ Baseline performance prior to noise exposure and hearing loss was estimated for 20 one-hour sessions over 2 weeks. After hearing loss and either VNS or Sham passive AT, all rats were tested for 20 additional sessions.

##### Tone detection testing

We also estimated each rat’s detection threshold for the 2 kHz target tone. The same psi-marginal adaptive procedure used in frequency discrimination testing was utilized to select the stimulus intensity on each trial, from 0 to 75 dB SPL in 3 dB steps. A 2 kHz tone was pseudorandomly presented on 50% of trials. The remaining 50% of trials were silent catch trials. Baseline performance prior to noise exposure and hearing loss was estimated for 10 one-hour sessions over 1 week. After hearing loss and either VNS or Sham passive AT, all rats were tested for 10 additional sessions.

##### Speech discrimination

After hearing loss and passive AT, and following re-estimation of psychometric thresholds, all rats were trained on a novel speech discrimination task using the same Go/No-go procedure. Rats had to discriminate the target speech syllable "Dad" from competing non-target syllables, "Bad", "Gad", "Tad", and "Sad". These stimuli have been described previously in other studies from our lab.^63^^,^^67^^,^^98^ Briefly, they were spoken by an adult female and shifted up by one octave using the STRAIGHT vocoder^100^ to match the higher frequency of rat hearing (see Figure S4, Experiment 1). They were calibrated such that the intensity of the loudest 100 ms of the vowel component of the sound was 60 dB SPL. Rats heard all speech sounds in every training session. Approximately 33% of trials pseudorandomly presented the target speech sound, "Dad", 33% presented one of the four non-target speech sounds (8.25% chance for each non-target sound), and the remaining trials were silent catch trials. Rats heard, on average, 240 ± 3 trials per day presenting the target speech sound, and 60 ± 1 trials per day presenting each of the non-target speech sounds (240 non-target trials in total). This presentation schedule allows rats to learn multiple speech discriminations within the task while maintaining adequate levels of motivation.^60^^,^^63^^,^^67^^,^^101^ While training, rats in the VNS group were stimulated during correct hits to the target speech sound (see "Active AT" in Methods). Rats trained on this task for 30 sessions over 3 weeks, and were then tested on a speech-in-noise version.

##### Speech-in-noise

The speech-in-noise task was identical to the speech discrimination task except with added speech-shaped background noise^60^^,^^64^ during blocks of trials. Silence, 54 dB SPL noise, or 60 dB SPL noise was pseudorandomly selected during a trial block and continuously played during behavior. The noise level changed every block of 20 trials, and could not be repeated in back-to-back blocks. Every session began with either 54 dB or 60 dB noise. Background noise was faded in and out using a 1 s linear ramp, and rats were given 10 seconds to adapt to every change in noise level before behavior sessions continued to the next trial. Rats were not plugged in and no VNS was delivered during speech-in-noise testing. Rats were tested for 10 sessions over 1 week.

#### Experiment 2

##### Speech training and testing

The speech tasks and methods for Experiment 2 have been published previously.^60^ Briefly, after noise-induced hearing loss and either VNS or Sham passive AT, all rats were tested on a speech detection task for 1 week. The target sound, "Shad", was presented at either 45, 60, or 75 dB SPL, calibrated to the loudest 100 ms of the sound. The target speech sound was pseudorandomly presented on 50% of trials. The remaining 50% of trials were silent catch trials. Rats were then trained over 5 weeks to discriminate "Shad" from competing non-target syllables, "Had", "Jad", and "Sad", followed by 1 week of testing. As with the detection task, speech sounds were presented at either 45, 60, or 75 dB SPL. The target speech sound, "Shad", was pseudorandomly presented on 50% of trials, one of the three non-target speech sounds were presented on 37% of trials, and the remaining trials were silent catch trials. After discrimination testing, rats trained on the same discrimination task with the addition of 60 dB SPL speech-shaped background noise^60^^,^^64^ for 2 weeks, followed by 1 week of speech-in-noise testing. The distribution of speech intensities in all tasks was 5% 45 dB SPL, 15% 60 dB SPL, and 80% 75 dB SPL. Details of the speech syllables used in Experiment 2 are identical to those described in Experiment 1 (see Figure S4, Experiment 2 for spectrograms).

#### Noise exposure

##### Experiment 1

After finishing baseline assessments for tone detection and discrimination, a subset of rats underwent noise-induced hearing loss, as done previously.^60^ Briefly, rats were given intraperitoneal injections of diazepam (5 mg/kg) 10 minutes prior to noise exposure to reduce stress and avoid audiogenic seizures. They were then placed in an 8.5″ × 4.5" × 6″ acoustically transparent wire cage on a rotating platform inside of a sound-attenuating custom acoustic enclosure. After 10 minutes of habituation to the space, rats were exposed to 1/4 octave broadband noise centered at 22 kHz at 120 dB SPL for 1 hour. These noise parameters were chosen based on pilot work which indicated that they reliably induce moderate low- and mid-frequency hearing loss and severe high-frequency hearing loss in rats. After noise exposure, rats were transferred back to their home cage and recovered for 3 weeks.

##### Experiment 2

In this experiment, rats underwent noise-induced hearing loss after reaching initial proficiency on speech detection and the basic Go/No-go procedure. The noise exposure procedure was identical to Experiment 1, except that the frequency and intensity parameters of the noise used varied across rats: 7 were exposed to noise with a 4 kHz center frequency at 120 dB SPL, 13 were exposed to noise with 16 kHz center frequency at 120 dB SPL, 8 were exposed to 22 kHz center frequency at 120 dB SPL, and 8 were exposed to 22 kHz center frequency at 125 dB SPL. These noise parameters can produce a more significant increase in ABR thresholds across the rat hearing range compared to the parameters used in Experiment 1.^60^ All noise exposures lasted for 1 hour. The first 10 rats in the experiment were randomly assigned to a set of NE parameters. All remaining rats were assigned to NE parameter sets which resulted in balanced pre-training performance levels between groups and which sought to minimize deaf or unimpaired rats. After noise exposure, rats were transferred back to their home cage and recovered for 4 weeks.

##### Vagus nerve cuff implant surgery

Three to four weeks after noise exposure, rats were implanted with a custom, platinum-iridium bipolar cuff electrode around the vagus nerve, as described previously.^41^^,^^61^^,^^102^ Briefly, rats were anesthetized using ketamine hydrochloride (80 mg/kg, intraperitoneal (IP) injection) and xylazine (10 mg/kg IP) and provided supplemental doses as needed to maintain anesthesia. A two-channel connector was affixed to the skull between the lambda and bregma suture landmarks using bonescrews and acrylic. The VNS cuff was secured around the left cervical vagus nerve and its two wire leads tunneled subcutaneously to the top of the head. The leads were then connected to the affixed skull connector, and a brief electrical stimulation was applied through the circuit to trigger the Hering-Breuer reflex, a quick cessation of breathing followed by a predictable drop in oxygen saturation.^103^ This reflex was used to validate the integrity of the electrode and cuff and verify that the vagus nerve was being stimulated. All incisions were then sutured closed, and the cuff leads and connector encapsulated with acrylic to protect the wires. Rats were given antibiotics and analgesics and then immediately moved to ABR testing while still anesthetized. Rats were monitored for 7 days after surgery to ensure a full and healthy recovery prior to continuing in the experiment.

##### Auditory brainstem response

Auditory brainstem responses (ABRs) were recorded for noise-exposed rats immediately following completion of cuff implant surgery, while they were still anesthetized. Hearing thresholds were estimated using 2, 4, 8, 16, and 32 kHz tonal ABRs presented in 10 dB steps from 5-95 dB SPL.^60^ Briefly, anesthetized rats were placed on a low-noise heating pad inside of a custom anechoic acoustic chamber lined with sound insulating foam. A calibrated free-field speaker (MF1, Tucker-Davis Technologies) was placed 10 cm from their left ear. Each frequency-intensity combination was repeated >1000 times, and the mean neural signal obtained through neurophysiology A/D hardware (RZ6 and BioSigRZ, or RZ5 and Brainware, Tucker-David Technologies). ABR thresholds for all tone frequencies were determined for each rat based on the minimum intensity at which a neural response was considered observable by blinded researchers (for example see Figure 1A, top). If no neural signal could be determined at any of the presented intensities, then the threshold was marked as being 85 dB SPL or greater (Figure 1A, bottom).

ABRs from normal hearing litter-mate controls (n = 64) were collected using identical procedures for comparison with noise-exposed rats. Following ABR collection, rats were randomized into VNS or Sham therapy groups such that mean ABR thresholds across frequencies were balanced between each set of VNS/Sham groups. In Experiment 1, rats were randomized into VNS (n = 14) and Sham (n = 13) therapy groups (Figure 1B). In Experiment 2, rats were first sorted into one of two hearing loss categories (Severe/Profound), and then further randomized into VNS (Severe n = 9, Profound n = 10) and Sham (Severe n = 12, Profound n = 5) therapy groups (Figure 6A). As done previously, rats were considered to have Profound hearing loss if their ABR thresholds across tested frequencies were all greater than 80 dB SPL, while NE rats that still maintained moderate low- or mid-frequency hearing were categorized as having Severe hearing loss.^60^

##### VNS parameters

Stimulation parameters were constant and consisted of a 500 ms train of 100 μs charge-balanced, biphasic pulses delivered at a current intensity of 0.8 mA and a frequency of 30 Hz (15 total pulses each train). These parameters are known to drive plasticity throughout the central nervous system across multiple domains and rodent models.^33^^,^^36^^,^^41^^,^^42^^,^^57^^,^^61^^,^^96^^,^^104^ A cable was plugged into rat skull connectors and attached to commutators on the ceiling of behavioral chambers, allowing freedom of movement throughout training and therapy. The commutators were connected to stimulators (A-M Systems, model 4100) to permit delivery of VNS. These stimulators deliver a highly consistent stimulus using the selected VNS parameters even with modest fluctuations in circuit impedance over time. Voltage waveforms in the VNS circuit were programmatically monitored online for artifacts in both the Passive and Active AT pairing sessions to ensure consistent delivery of stimulation. Spikes in voltage readings would pause running sessions and alert researchers to investigate any issues. Any rat determined to have a broken cuff or peak-to-peak voltage measurement over 20 V was marked as a VNS cuff failure and removed from the study and analyses.

##### AT Passive

Following recovery from cuff implant surgery, NE rats underwent 20 sessions of passive AT over a 4 week period.^41^^,^^42^^,^^61^ Sessions were 2.5 hours long, 1 session per day, 5 days per week. Rats in the VNS group were plugged into stimulators and placed in a 25 cm × 25 cm x 25 cm acoustically transparent wire cage inside of a double-walled sound attenuation chamber lined with acoustic insulating foam. During therapy sessions, sounds were presented from a speaker mounted above the cage and paired with delivery of VNS. The inter-stimulus interval between VNS-sound pairings was 30.85 ± 22.11 s (mean ± SD) with a minimum interval of 15 s. VNS pairings occurred 300 times per day. Rats in the Sham group were brought out of the animal housing room and passively exposed to laboratory sounds, but did not receive VNS or targeted sound presentations.

In Experiment 1, VNS was paired with the presentation of a 500 ms 2 kHz 60 dB SPL tone, with stimulation onset preceding tone onset by 150 ms. In Experiment 2, VNS was paired with the presentation of the speech sound "Shad", with stimulation onset preceding speech onset by 50 ms. These separate offset timings have been repeatedly used for VNS pairing with either tones or speech, respectively, and were selected to ensure significant overlap of nerve stimulation with presentation of the paired auditory stimulus.^41^^,^^61^^,^^89^ However, it is worth noting that previous reports show stimulus offsets between −200 to +50 ms from sound onset result in indistinguishable levels of plasticity.^41^^,^^105^ Experiment 2 also altered the intensity of "Shad" presentations throughout the therapy timeline. The first therapy session initially presented "Shad" calibrated to 75 dB SPL. Every subsequent session reduced the initial presentation intensity by 1 dB, lowering to 55 dB in the final therapy session. Within sessions, the intensity lowered by 0.1 dB SPL every third stimulation, gradually lowering 10 dB by the end of each session. VNS was thus paired with "Shad" presentations ranging from 75 to 45 dB SPL over the course of therapy.

##### Active AT (Experiment 1 only)

NE rats from both VNS and Sham groups were plugged into stimulators via their surgically implanted skull connectors during speech discrimination training. Rats in the VNS group received stimulation paired with correct responses to the target speech sound, "Dad", a stimulation strategy that has repeatedly improved recovery in deficit models^33^^,^^34^^,^^38^^,^^104^ and may augment reinforcement signals during learning.^56^ VNS rats received 197.3 ± 5.7 (mean ± sem) VNS pairings per day. Rats in the Sham group did not receive any stimulation. Programmatic, online voltage monitoring was performed to ensure rats received stimulation appropriately, but researchers running behavior were otherwise blind to therapy group membership.

##### Timeline

See Figure S1 for schematic timelines for each experiment.

### Quantification and statistical analysis

Analyses were performed blinded using custom R scripts. We employed Restricted Maxiumum Likelihood linear mixed models with individual rats as random effects using the lme4 R package,^106^ as these models tend to be robust to violations of statistical assumptions and work well with unequal group sizes.^107^ We used the lmerTest package^108^ to calculate p values of our fixed effects using F-tests with degrees of freedom estimated by Satterthwaite approximations. These tend to produce acceptable Type I error rates even for unbalanced datasets.^109^ Post-hoc contrasts of the estimated marginal means were calculated using the emmeans package^110^ with Kenward-Roger estimations of degrees of freedom. Corrections for multiple comparisons were performed using the multivariate **t** distribution method.

To complement our linear mixed model analyses, supplementary analyses using Bayesian hierarchical models are included for our main speech discrimination results (Figures 4, 5, 6, and S13–S18). Bayesian analyses are robust in contexts with unbalanced group sizes and low sample numbers, as uncertainties in parameter estimates are propagated through the model and reflected in the results. The output of these analyses are also simple probability distributions and do not require corrections for multiple comparisons, allowing for easy interpretation of contrasts.^111^^,^^112^ We used the rstan and brms R packages^113^^,^^114^^,^^115^ to fit the models and the tidybayes and emmeans^110^^,^^116^ R packages for comparison of model estimates and visualizations. Performance data were modeled as normal distributions with a broad prior distribution centered at chance performance (50% correct). Error parameters were given exponential prior distributions with rates of 0.125 (corresponding to an expected ±8% discrimination performance error). Population effects, such as VNS or Sham treatment, were given normal prior distributions with a mean of 0 and a standard deviation of 10, a specification broad enough to permit individual effects to fully explain possible differences in discrimination performance and narrow enough to constrain results within the limits of the behavioral task (e.g., rats cannot be less than 0% or more than 100% correct on speech discrimination). Models were sampled 20,000 times each on 4 MCMC chains with a 4,000 sample burn-in. All parameters achieved an effective bulk and tail sample size of at least 10,000 and converged with diagnostic statistic R-hat <1.01, indicating reliable posterior estimates.^117^ We found these supplementary Bayesian analyses corroborate the results and conclusions derived from our linear mixed model analyses, and so only the linear mixed model results are included in the main text for clarity.

ABRs which did not contain an observable threshold had imputed values of 95 dB for statistical comparisons. A supplementary set of ABR threshold survival analyses (Figures S2 and S5) using Kaplan-Meier survival curves and censored data indicators instead of imputed values corroborate our ABR results reported in the main text (survival R package^118^). All VNS subjects were re-tested for VNS elicited triggering of the Hering-Breuer reflex under anesthesia after finishing the experiment to ensure the implanted cuff was still functional. Any subject which did not demonstrate a reflex response was considered to have a VNS cuff failure and was removed from all final analyses. Figures S7–S12 show individual data for each of the corresponding figures in the main text. Table S1 contains an aggregate of all reported statistical comparisons, organized by associated figure panels.

## Supplemental information

Document S1. Figures S1–S18

Table S1. Aggregated statistical comparisons, related to STAR Methods
