# Supplementary material for: Bursts of vagus nerve stimulation paired with auditory rehabilitation fail to improve speech sound perception in rats with hearing loss
Source: iScience. 2024 Mar 19;27(4):109527. doi: 10.1016/j.isci.2024.109527 (PMC10995867; doi:10.1016/j.isci.2024.109527)
Supplement: Document S1. Figures S1–S18 [file mmc1.pdf]

iScience, Volume *27*

Supplemental information

Bursts of vagus nerve stimulation paired with auditory rehabilitation fail to improve speech sound perception in rats with hearing loss

Alan M. Carroll, Jonathan R. Riley, Michael S. Borland, Tanya T. Danaphongse, Seth A. Hays, Michael P. Kilgard, and Crystal T. Engineer

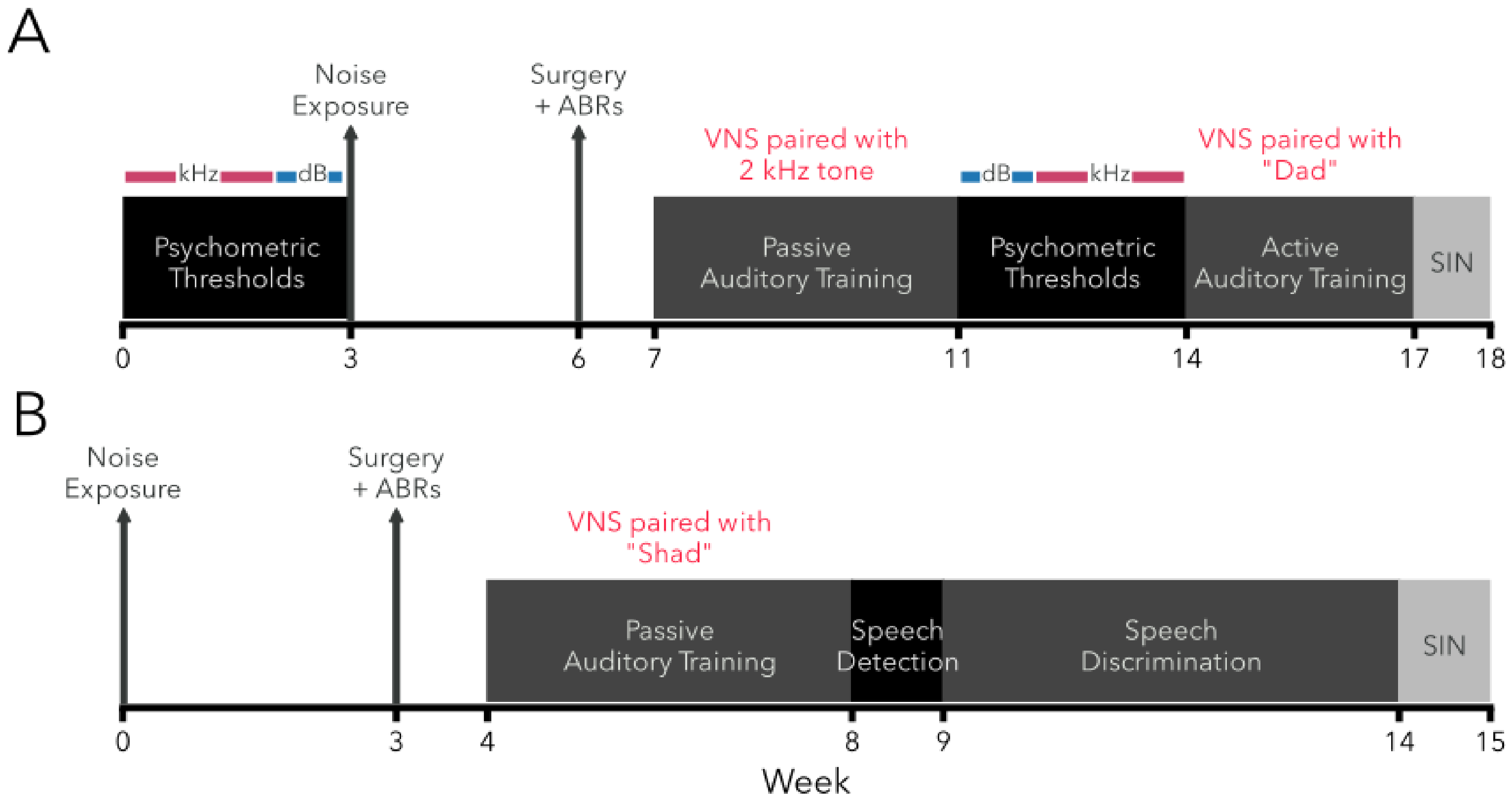

Figure S1: Related to STAR Methods. Experiment timelines. (**a**) Experiment 1. Psychometric Threshold testing includes Tone Detection (dB) and Tone Discrimination (kHz). (**b**) Experiment 2.

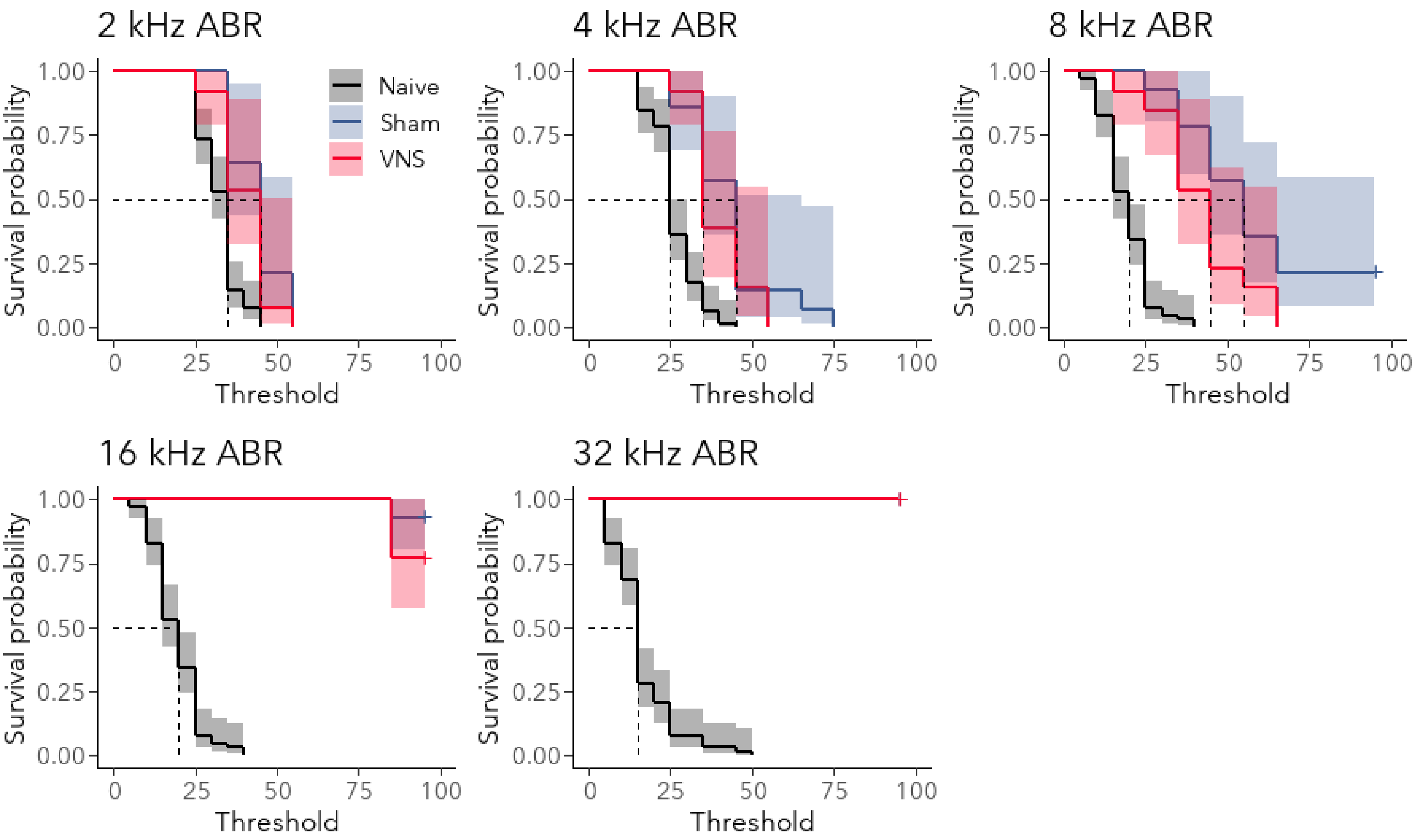

Figure S2: Related to Figure 2. Fitted Kaplan-Meier survival curves of ABR thresholds in Experiment 1 for each of the tested tone frequencies. Shaded regions show 95% confidence intervals.

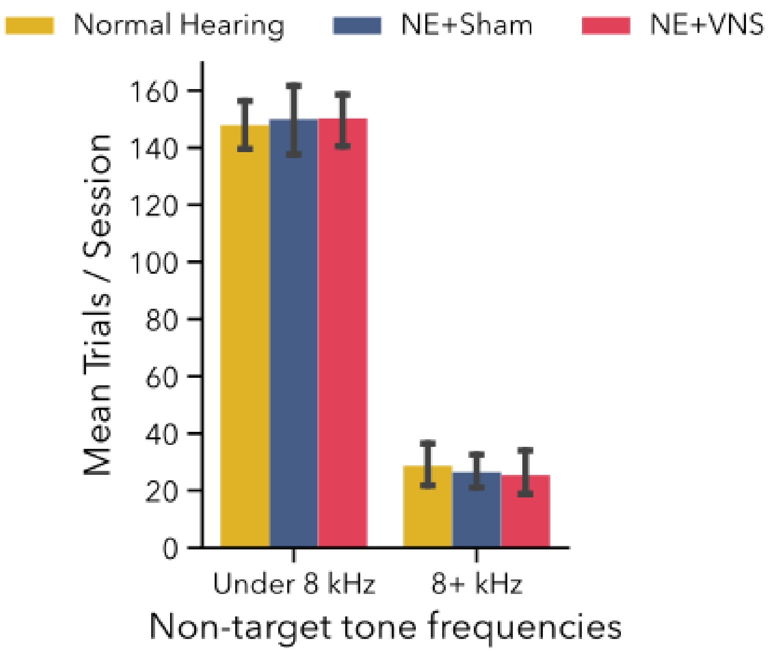

Figure S3: Related to Figure 3. Proportion of trials presenting tones under and over 8 kHz during tone discrimination in Experiment 1 (mean ± sem).

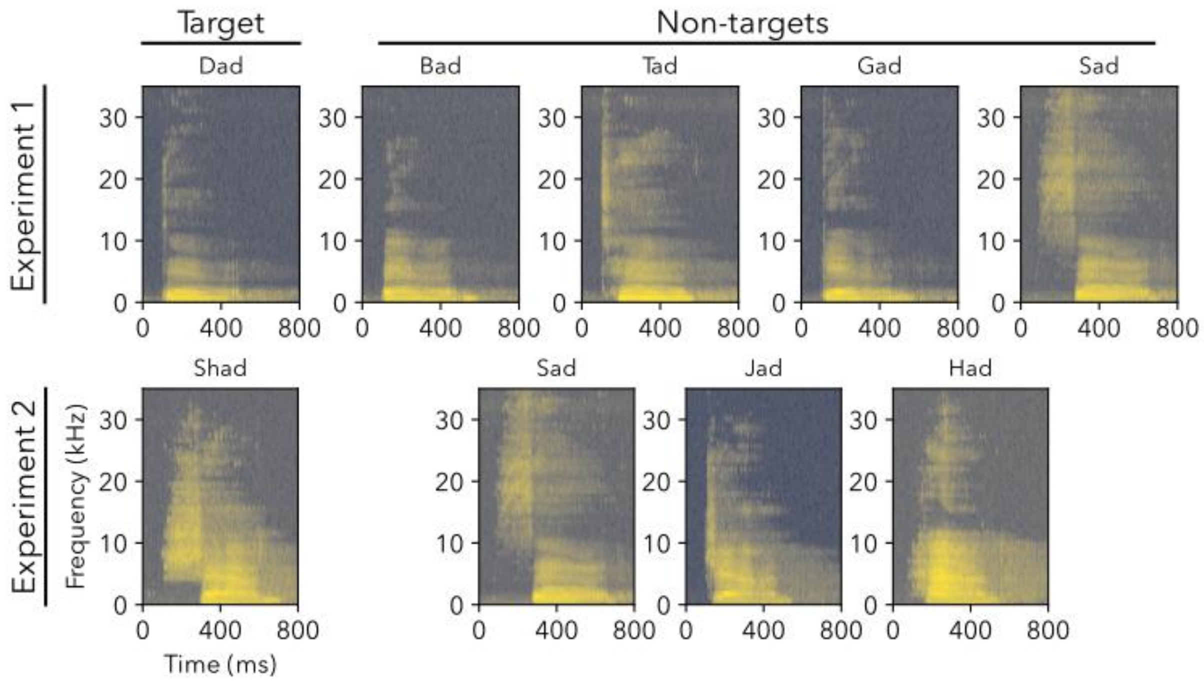

Figure S4: Related to Figure 4 and 6. Spectrograms of the first 800 ms of speech sounds used in this study. Sounds were shifted one octave higher to accommodate the rat hearing range.

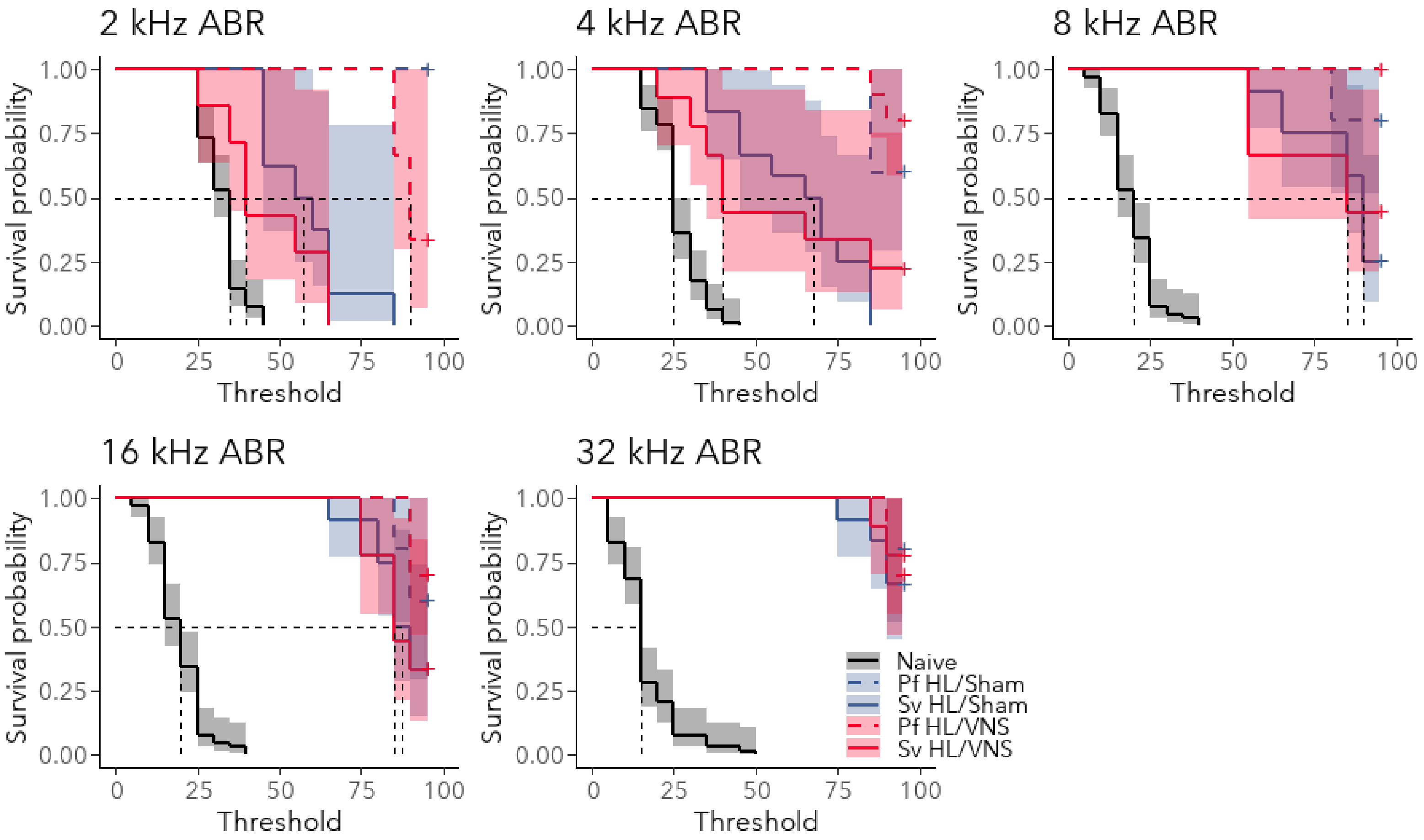

Figure S5: Related to Figure 6. Fitted Kaplan-Meier survival curves of ABR thresholds in Experiment 2 for each of the tested tone frequencies. Shaded regions show 95% confidence intervals.

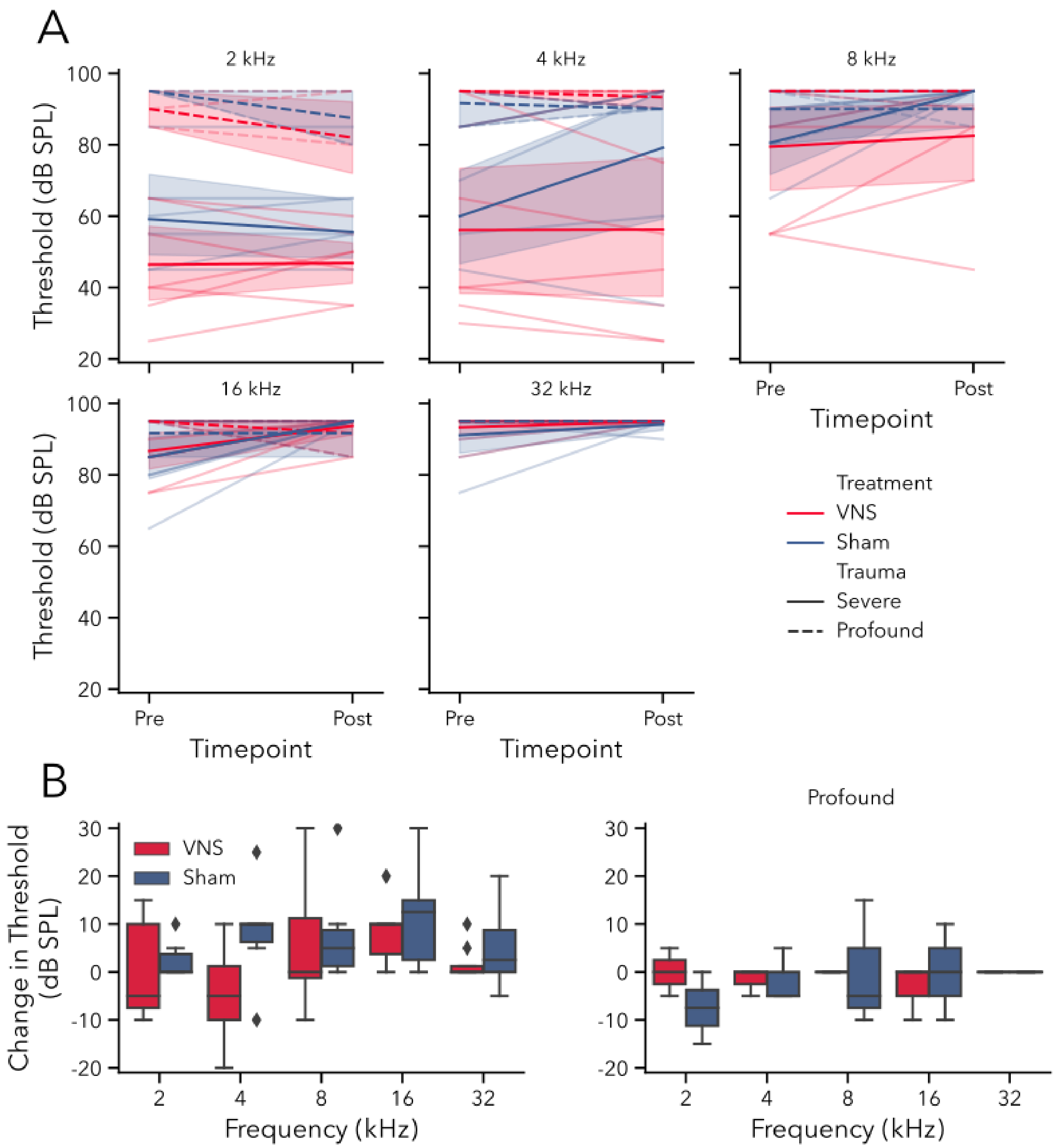

Figure S6: Related to Figure 6. Experiment 2, change in noise-exposed ABR thresholds after VNS or Sham treatment and speech testing. Pre-treatment thresholds were measured 3 weeks after noise-induced hearing loss, and Post-treatment thresholds were measured at the end of behavior (see Supplementary Figure S1, bottom). No significant differences between VNS or Sham treatment were observed for either hearing loss group. **(a)** Pre- and Post-Treatment ABR thresholds. Bold lines and shaded regions show group means and 95% confidence intervals. **(b)** Change in threshold between Pre- and Post-Treatment timepoints (mean ± sem).

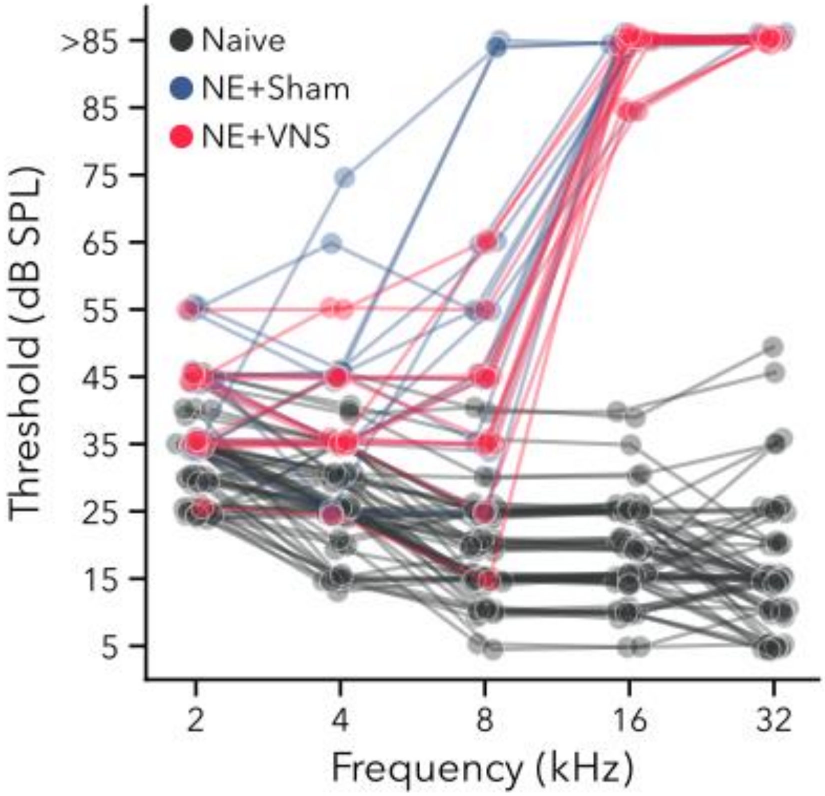

Figure S7: Related to Figure 1. Each line shows ABR thresholds for an individual rat.

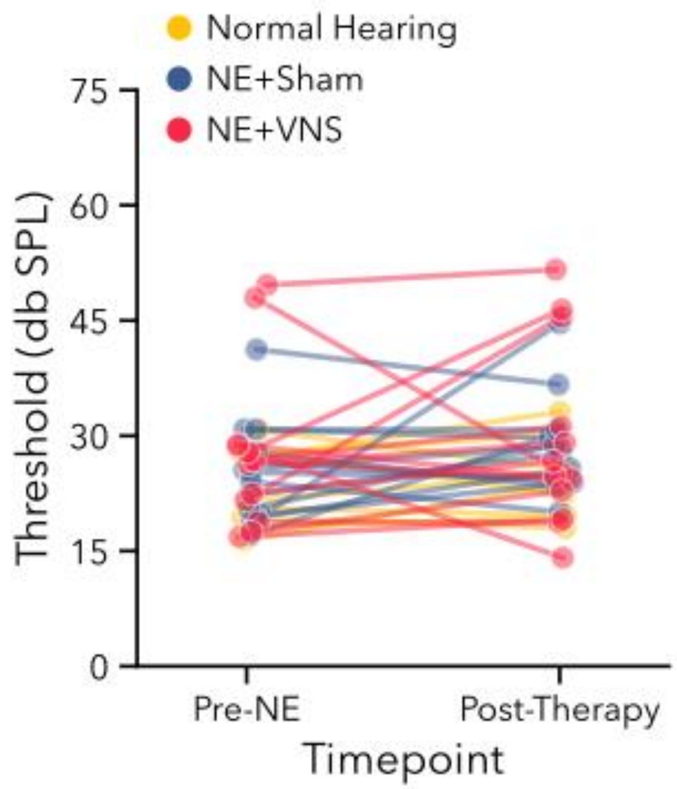

Figure S8: Related to Figure 2. Each line shows tone detection thresholds for an individual rat.

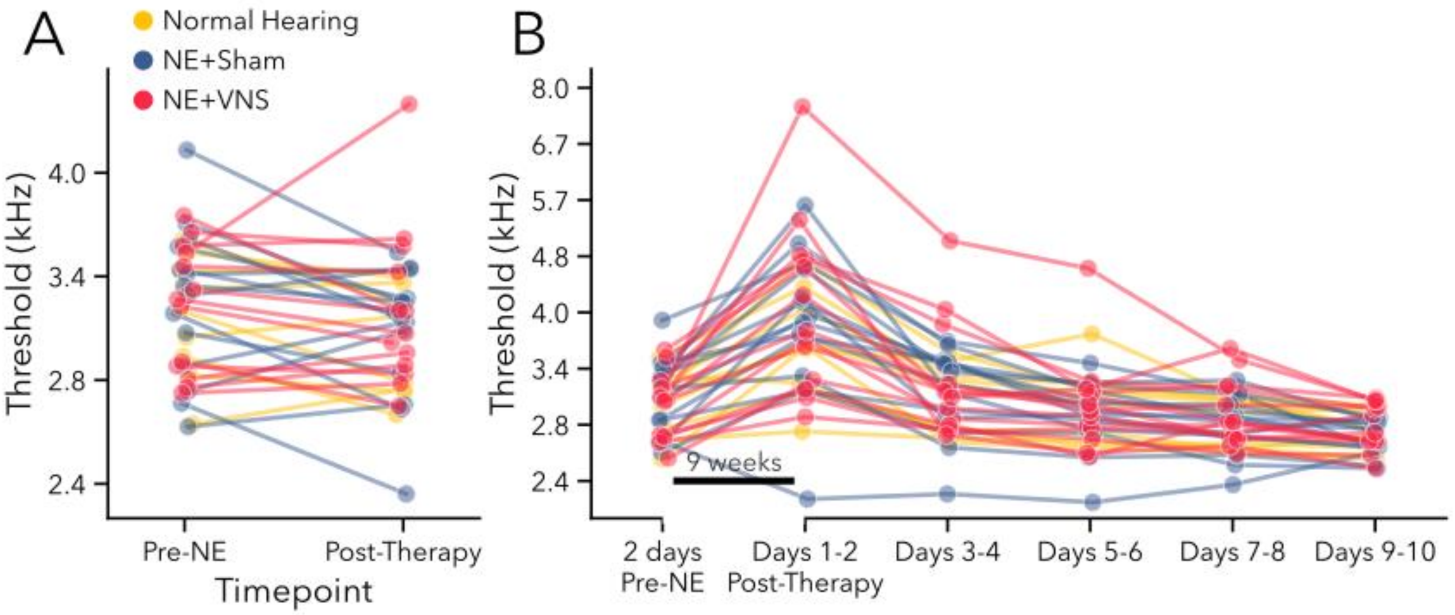

Figure S9: Related to Figure 3. Each line shows tone discrimination thresholds for an individual rat.

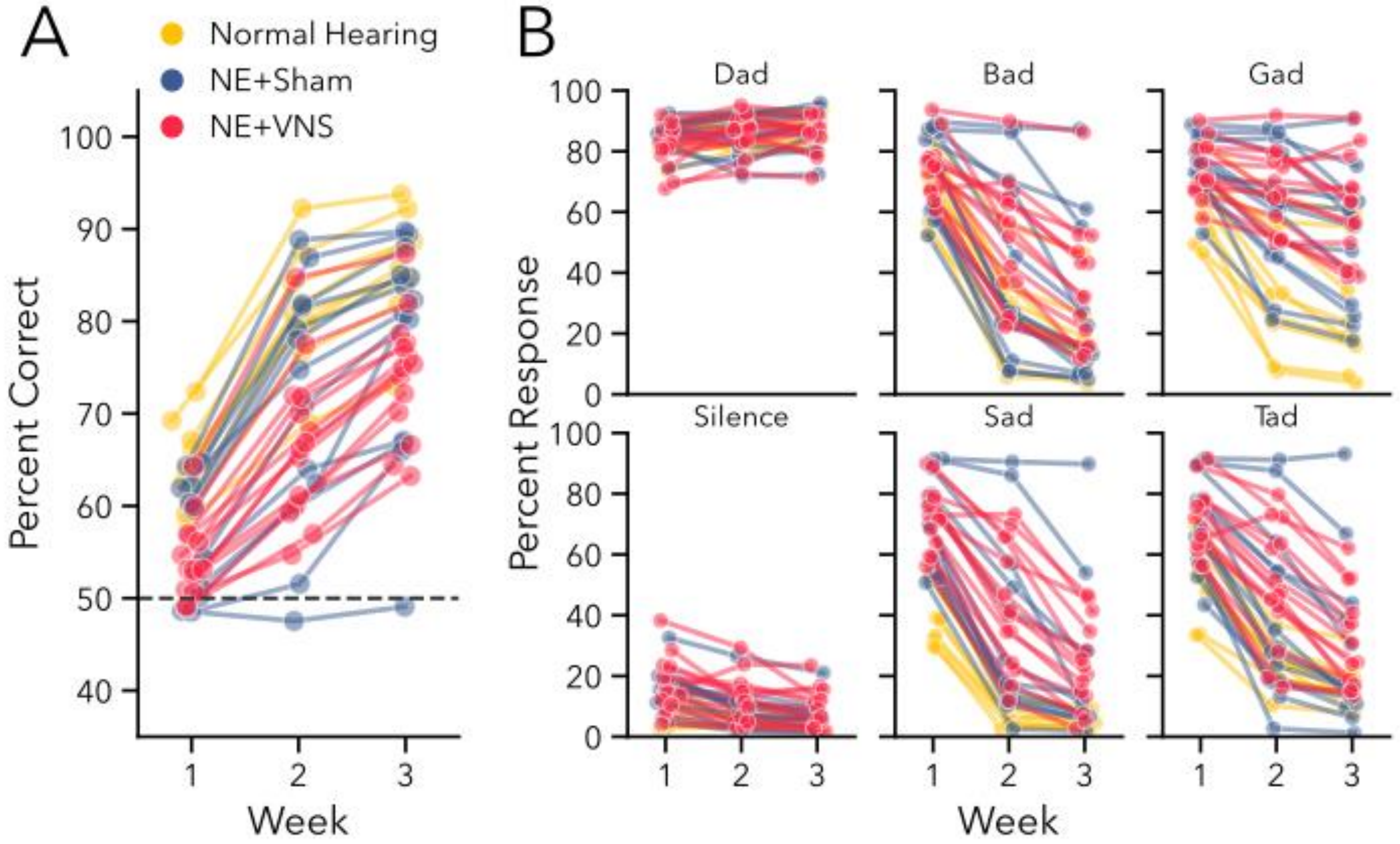

Figure S10: Related to Figure 4. Each line shows an individual rat’s speech discrimination performance.

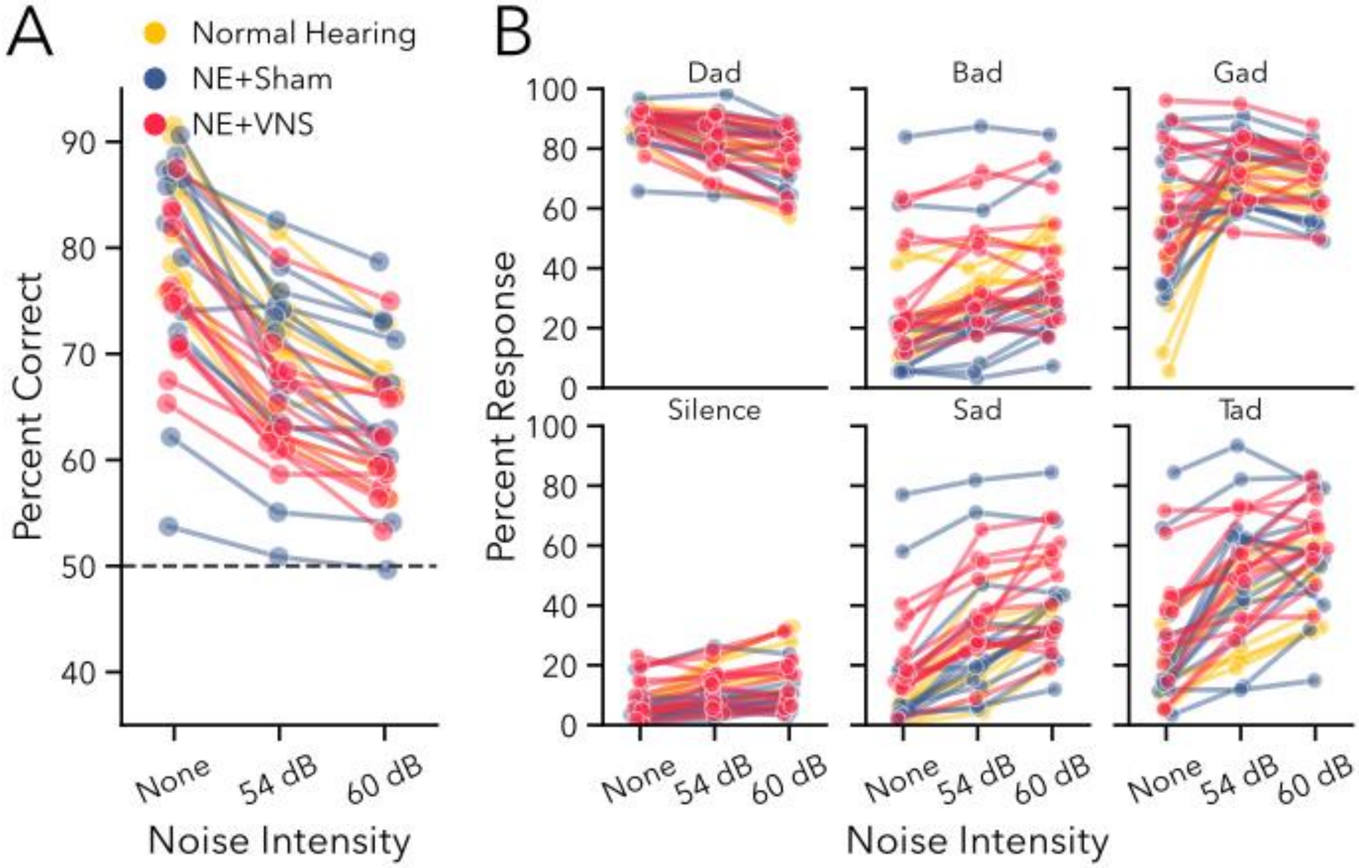

Figure S11: Related to Figure 5. Each line shows an individual rat's speech-in-noise discrimination performance.

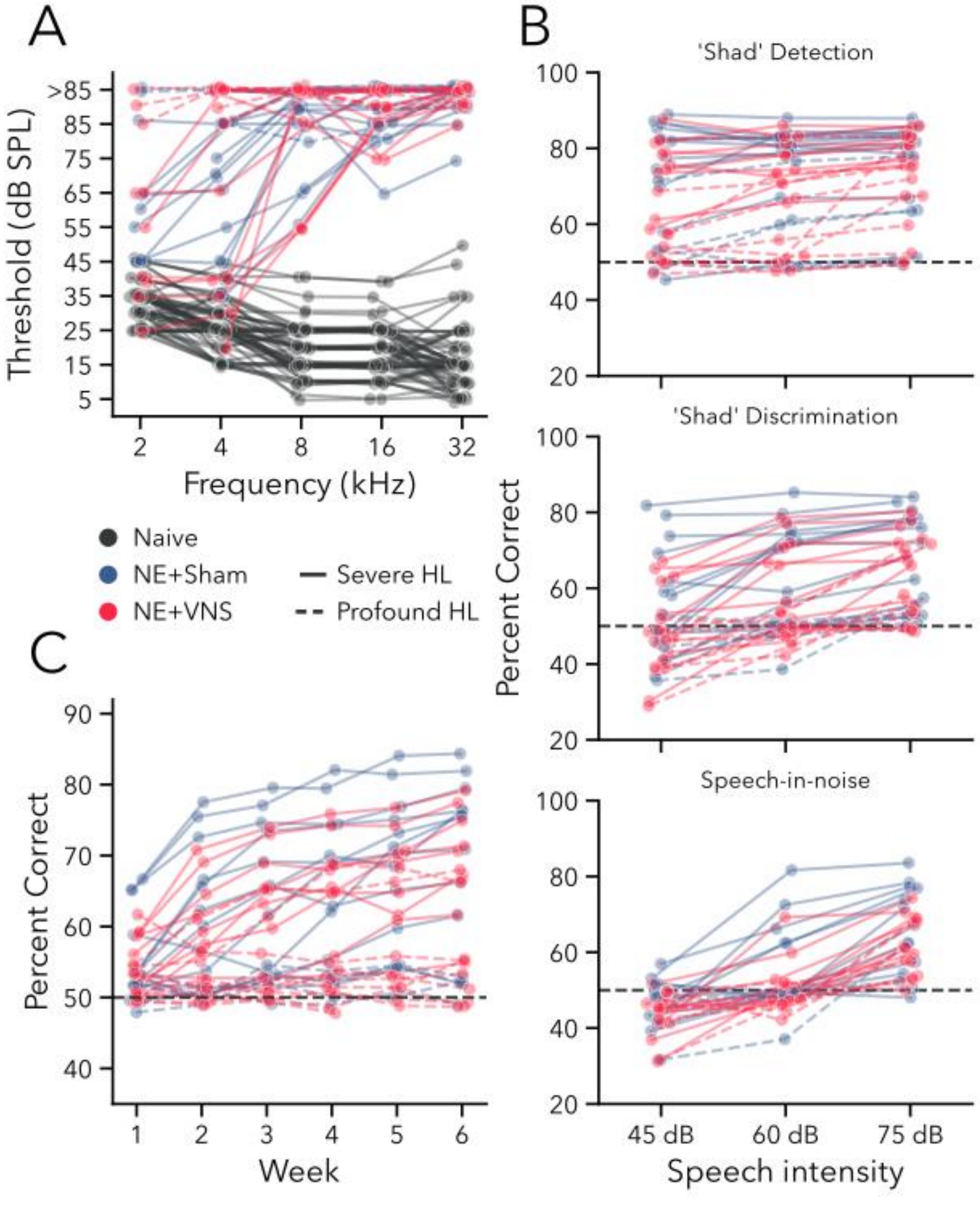

Figure S12: Related to Figure 6. Each line shows an individual rat's ABR thresholds **(a)**, speech detection/discrimination performance by speech intensity **(b)**, or speech discrimination performance over six weeks of training **(c)**.

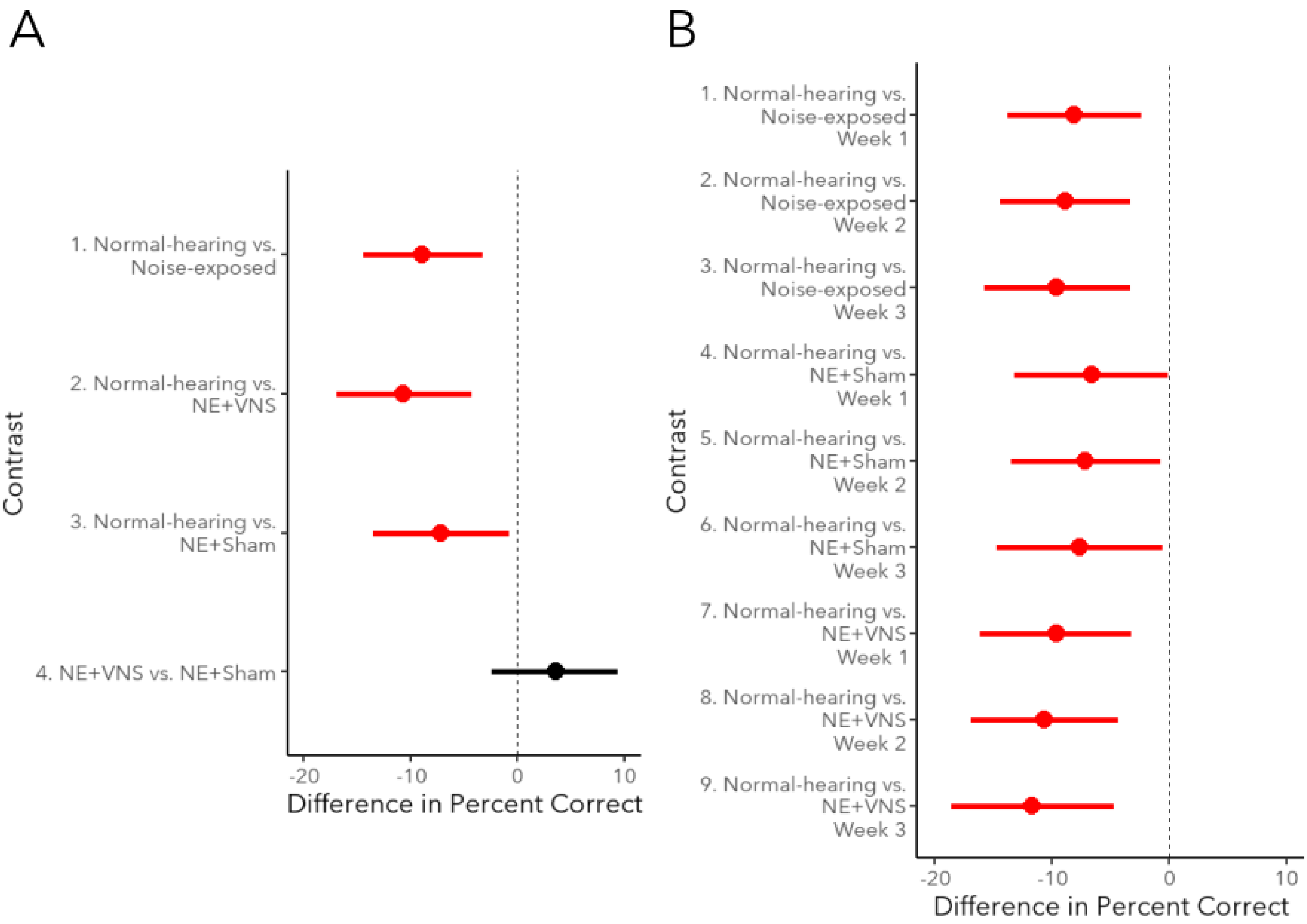

Figure S13: Related to Figure 4. Results of Bayesian analysis of Experiment 1 speech discrimination data. Each interval shows the 95% highest posterior density of the corresponding contrast and its median. Conditions which exclude the possibility of 0 difference to performance (Percent Correct) are highlighted red and considered statistically significant. Consistent with the frequentist analysis in the main text using a linear mixed-model, we find that noise exposure decreased performance compared to Normal-hearing control rats **(a, Contrast 1-3)**, and that VNS did not improve performance compared to Sham **(a, Contrast 4)**. Contrasting performance for each week shows that this performance deficit was consistent for noise-exposed rats overall **(b, Contrast 1-3)** and was not specific to either the Sham **(b, Contrast 4-6)** or VNS **(b, Contrast 7-9)** treatment groups.

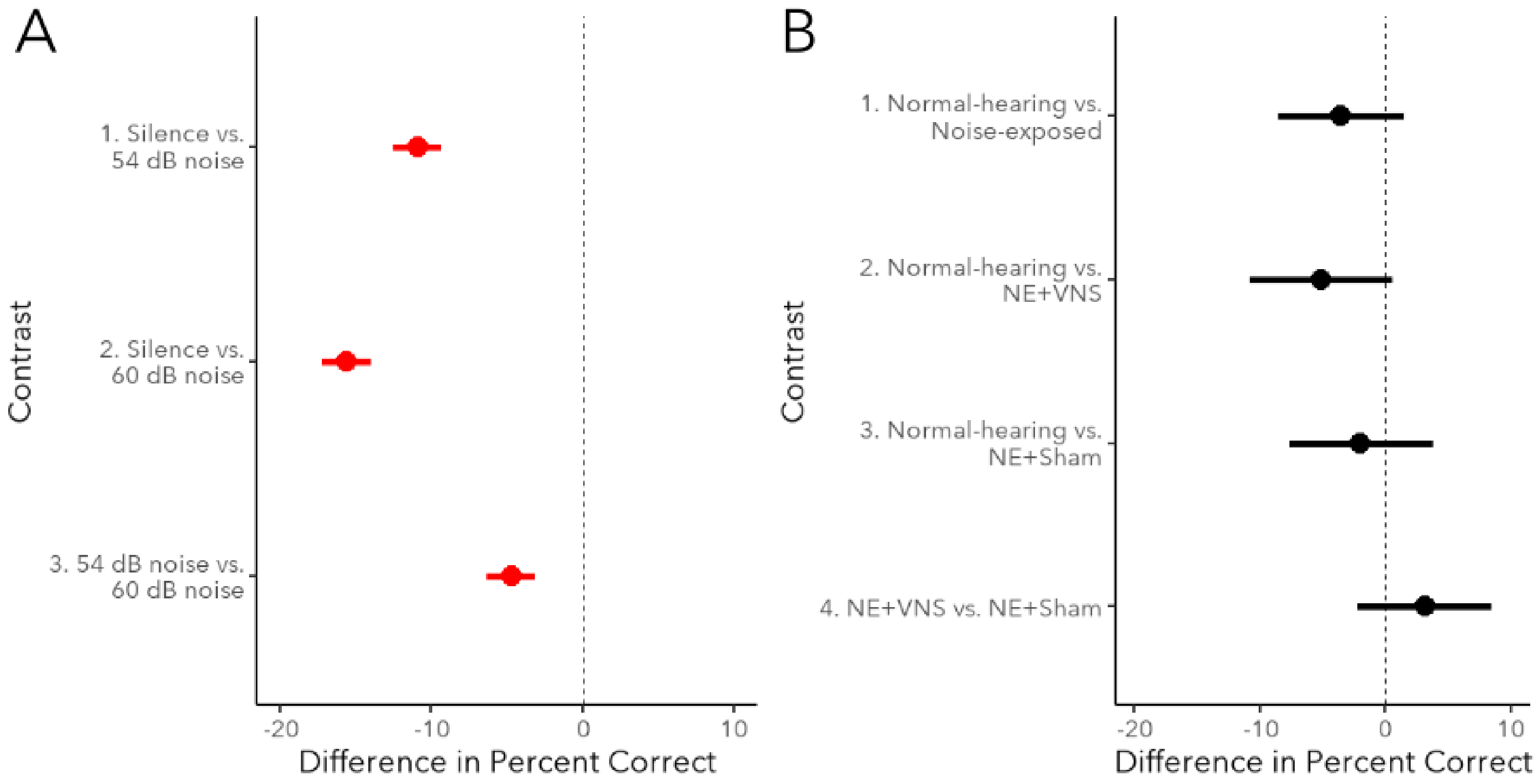

Figure S14: Related to Figure 5. Results of Bayesian analysis of Experiment 1 speech-in-noise discrimination data. Each interval shows the 95% highest posterior density of the corresponding contrast and its median. Conditions which exclude the possibility of 0 difference to performance (Percent Correct) are highlighted red and considered statistically significant. Consistent with the frequentist analysis in the main text using a linear mixed-model, we find that the addition of background noise decreased speech discrimination performance **(a, Contrast 1 and 2)**, rats performed worse with increasing noise levels **(a, Contrast 3)**, that hearing loss did not result in a performance deficit compared to Normal-hearing controls **(b, Contrast 1-3)**, and that VNS treatment did not drive performance improvements compared to Sham treatment **(b, Contrast 4)**.

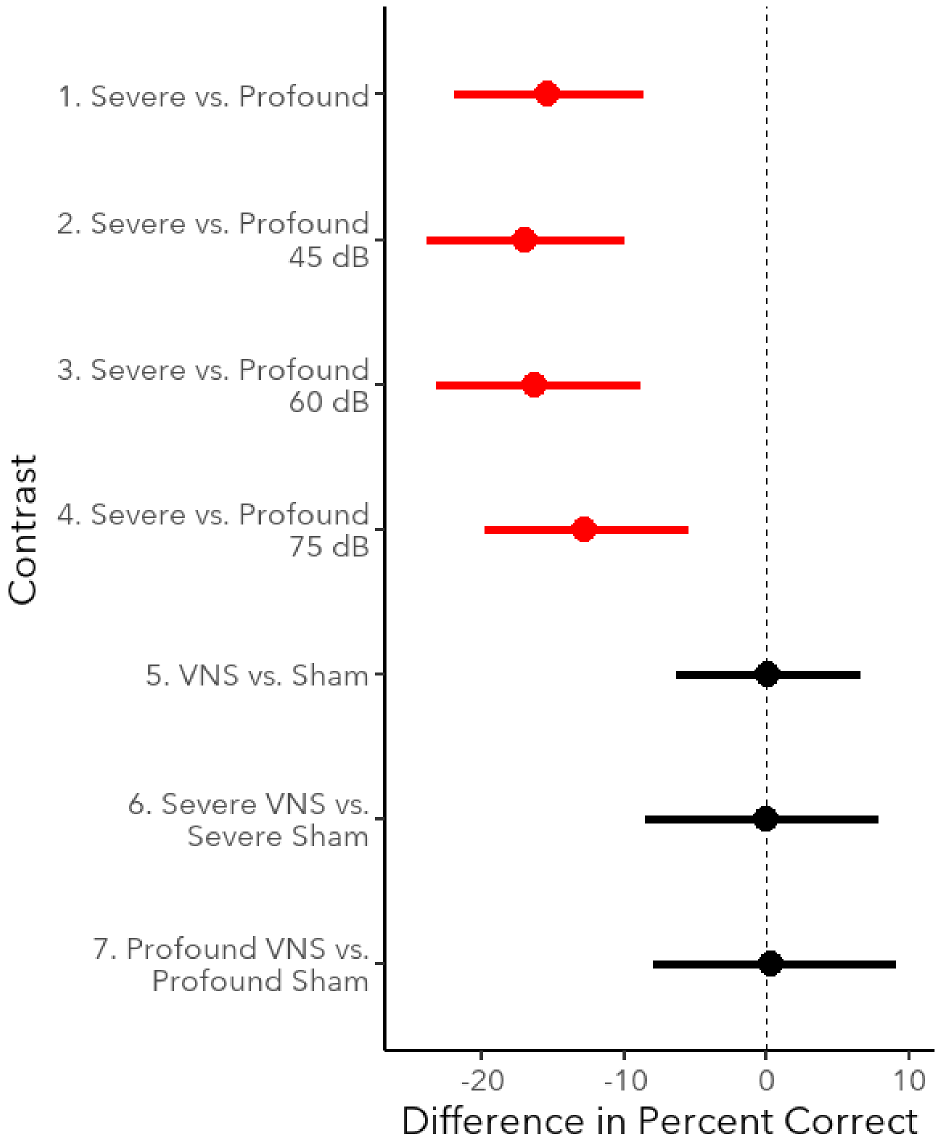

Figure S15: Related to Figure 6. Results of Bayesian analysis of Experiment 2 ‘Shad’ Detection data. Each interval shows the 95% highest posterior density of the corresponding contrast and its median. Conditions which exclude the possibility of 0 difference to performance (Percent Correct) are highlighted red and considered statistically significant. Consistent with the frequentist analysis in the main text using a linear mixed-model, we find that rats with Profound hearing loss performed worse than rats with Severe hearing loss both overall and across all speech intensity levels **(Contrast 1-4)**, and that VNS treatment did not improve performance compared to Sham treatment overall **(Contrast 5)** or for either hearing loss condition **(Contrast 6 and 7)**.

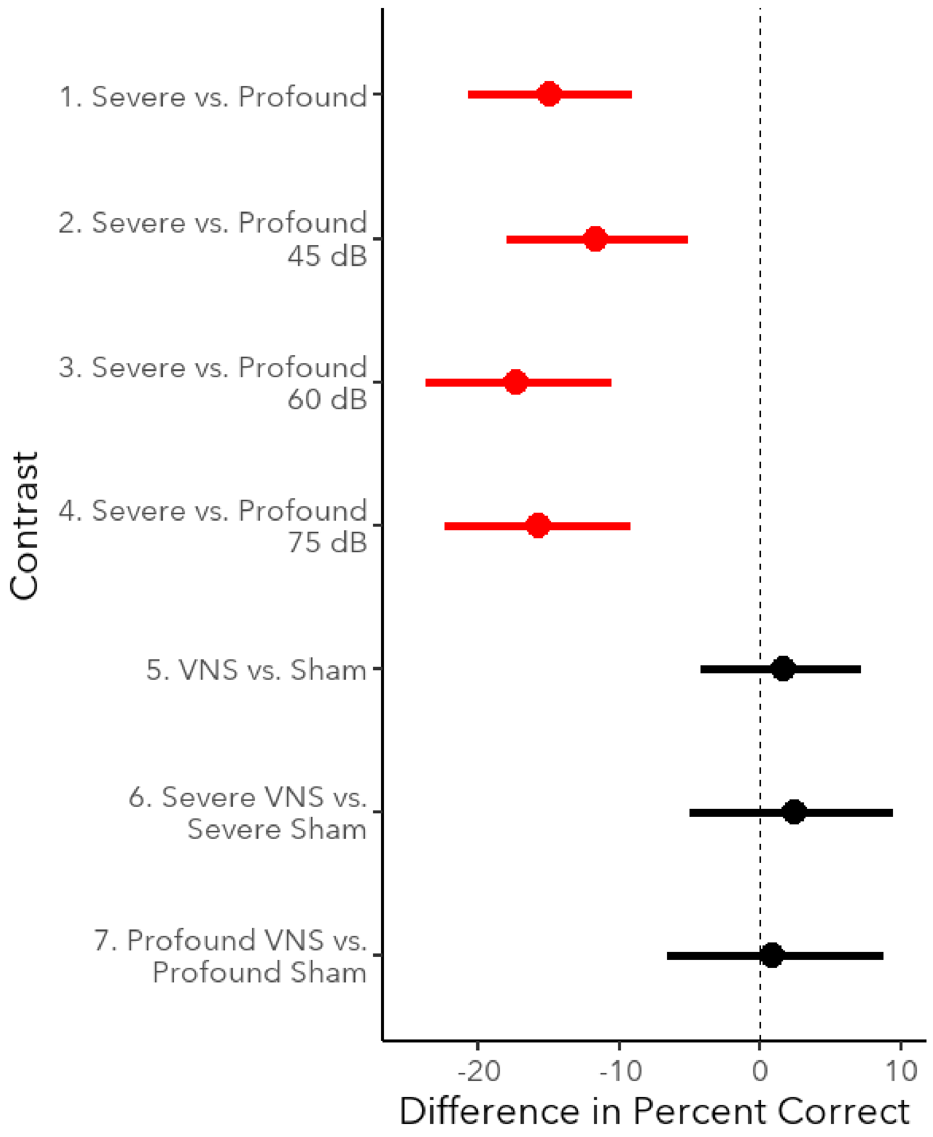

Figure S16: Related to Figure 6. Results of Bayesian analysis of Experiment 2 'Shad' Discrimination data. Each interval shows the 95% highest posterior density of the corresponding contrast and its median. Conditions which exclude the possibility of 0 difference to performance (Percent Correct) are highlighted red and considered statistically significant. Consistent with the frequentist analysis in the main text using a linear mixed-model, we find that rats with Profound hearing loss performed worse than rats with Severe hearing loss both overall and across all speech intensity levels **(Contrast 1-4)**, and that VNS treatment did not improve performance compared to Sham treatment overall **(Contrast 5)** or for either hearing loss condition **(Contrast 6 and 7)**.

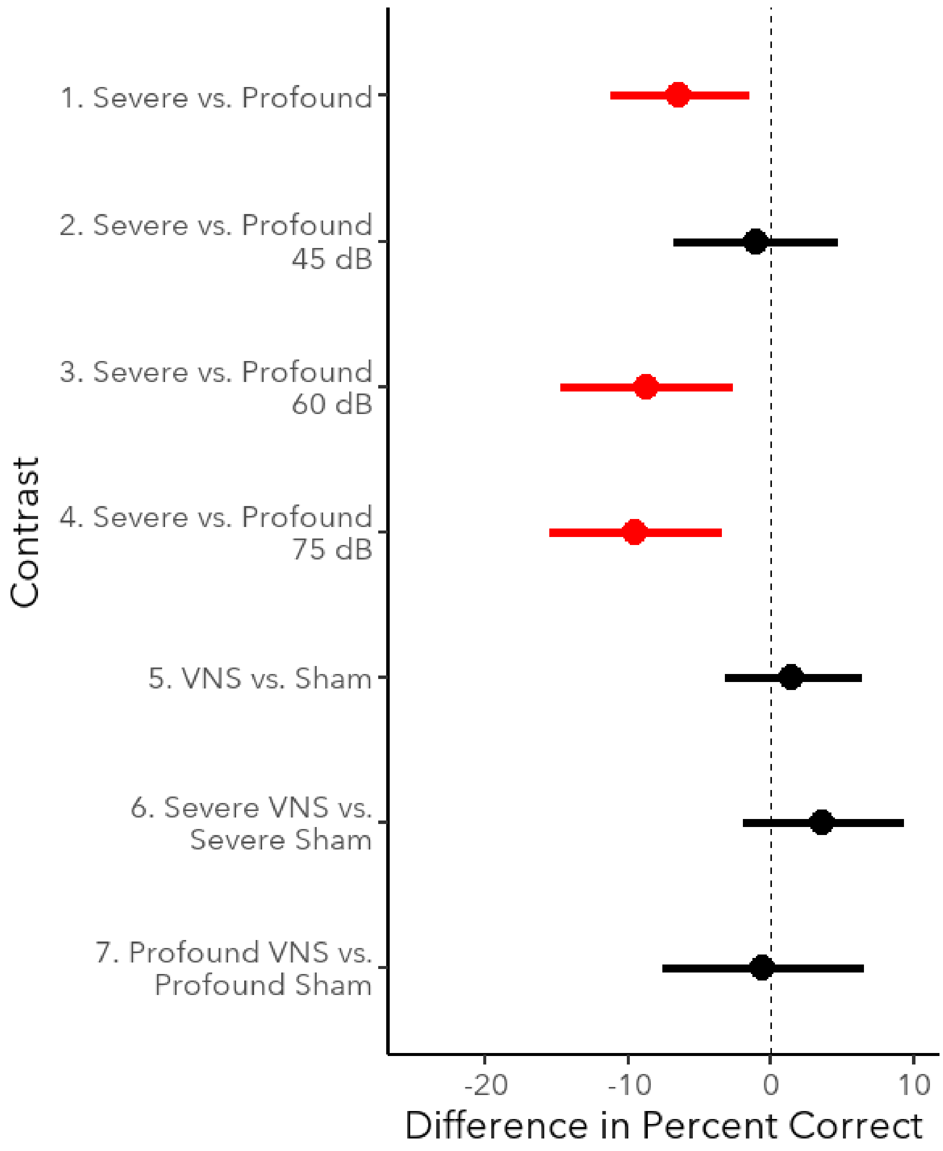

Figure S17: Related to Figure 6. Results of Bayesian analysis of Experiment 2 Speech-in-noise data. Each interval shows the 95% highest posterior density of the corresponding contrast and its median. Conditions which exclude the possibility of 0 difference to performance (Percent Correct) are highlighted red and considered statistically significant. Consistent with the frequentist analysis in the main text using a linear mixed-model, we find that rats with Profound hearing loss performed worse than rats with Severe hearing loss overall **(Contrast 1)** and for 60 and 75 dB speech intensities **(Contrast 3 and 4)**, and that VNS treatment did not improve performance compared to Sham treatment overall **(Contrast 5)** or for either hearing loss condition **(Contrast 6 and 7)**.

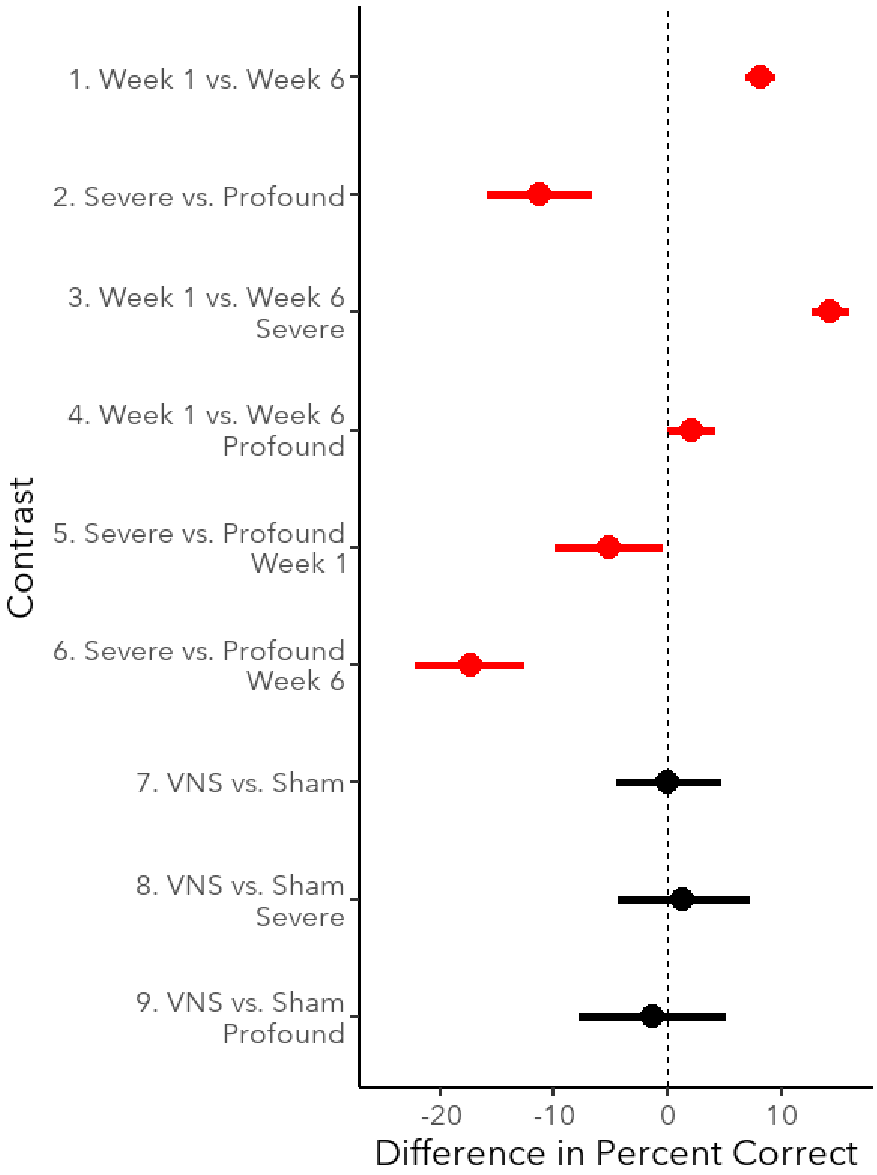

Figure S18: Related to Figure 6. Results of Bayesian analysis of Experiment 2 ‘Shad’ discrimination data over 6 weeks of training. Each interval shows the 95% highest posterior density of the corresponding contrast and its median. Conditions which exclude the possibility of 0 difference to performance (Percent Correct) are highlighted red and considered statistically significant. Consistent with the frequentist analysis in the main text using a linear mixed-model, we find that rats improved on speech discrimination over time **(Contrast 1)** and that rats with Profound hearing loss performed worse than Severe hearing loss overall **(Contrast 2)**. Both groups of rats performed better by the end of training **(Contrast 3 and 4)**, though rats with Severe hearing loss improved significantly more than rats with Profound hearing loss **(Contrast 5 and 6)**. VNS treatment did not improve performance compared to Sham treatment overall **(Contrast 7)** or for either hearing loss condition **(Contrast 8 and 9)**.
